# A study on the temporal network characteristics of early post-chemotherapy core symptoms in colorectal cancer patients based on ecological momentary assessment

**DOI:** 10.3389/fonc.2026.1942076

**Published:** 2026-10-27

**Authors:** Yuanfeng Lv, Longke Xu, Minxiang Li, Xiaoxia Cai, Lixiang Ye, Xinyu Wang, Xiaoshan Wang, Yanjing Chen, Yani Lu

**Affiliations:** 1 Hainan Medical University, School of Nursing, Haikou, China; 2 The Second Affiliated Hospital of Hainan Medical University, Nursing Department, Haikou, China

**Keywords:** chemotherapy, colorectal cancer, dynamic symptom network, ecological momentary assessment, linear mixed-effects model, nursing

## Abstract

**Background:**

Patients with colorectal cancer (CRC) often experience multiple concurrent symptoms during the early post-chemotherapy period, and both symptom severity and inter-symptom associations may change over time. Previous low-frequency assessments may not adequately capture short-term fluctuations.

**Objective:**

To characterize the dynamic trajectories and network features of early symptoms in patients receiving postoperative adjuvant chemotherapy for CRC and to identify candidate symptoms for enhanced monitoring.

**Methods:**

A prospective longitudinal study was conducted. Ecological momentary assessment (EMA) was performed three times daily for seven consecutive days after infusion in the first and second chemotherapy cycles and covered 13 symptoms. Linear mixed-effects models characterized symptom trajectories, and a multilevel vector autoregressive (mlVAR) model estimated temporal and contemporaneous networks. Edge uncertainty, centrality stability, sensitivity analyses, and between-cycle differences were evaluated.

**Results:**

A total of 110 patients contributed 4,134 valid assessments (completion rate, 89.5%). All 13 symptoms showed significant time effects. Fatigue, loss of appetite, gastrointestinal symptoms, and sleep disturbance fluctuated, whereas numbness/tingling and depression showed cumulative trends. Loss of appetite, dry mouth, and nausea had comparatively high outgoing temporal associations. The strongest individual temporal edge was modest (standardized coefficient approximately 0.12). No statistically significant between-cycle differences were found in overall network structure or global strength.

**Conclusion:**

Early post-chemotherapy symptoms showed dynamic changes and a broadly comparable network structure across the first two cycles. Loss of appetite, dry mouth, and nausea may be candidates for enhanced monitoring and future intervention research; the observational findings do not establish causal effects or intervention efficacy.

## 1 Introduction

Colorectal cancer (CRC) is a malignancy with both high incidence and mortality worldwide. According to 2024 data from the WHO International Agency for Research on Cancer (IARC) (1), new CRC cases globally accounted for approximately 10.2% of all incident cancer cases in 2022, ranking third in incidence, while CRC-related deaths represented about 9.3% of all cancer-related deaths, ranking second in mortality. At present, postoperative adjuvant chemotherapy is an essential treatment modality for reducing recurrence risk and improving prognosis, with approximately 90% of patients requiring three to six months of adjuvant chemotherapy (2). However, patients undergoing chemotherapy frequently experience multiple symptoms, including nausea, loss of appetite, sleep disturbance, and emotional distress (3). These symptoms may occur individually or co-occur as a symptom cluster, exhibiting marked dynamic changes across different phases of chemotherapy (4, 5). Persistent or recurrently exacerbated symptom burden not only significantly impairs patients’ functional status and quality of life, but also compromises treatment adherence and clinical prognosis, increases the risk of rehospitalization, and shortens survival (6–8). Therefore, understanding the changes and interactions of early post-chemotherapy symptoms in CRC patients is of great significance for optimizing symptom management strategies and improving the quality of nursing care.

Traditional symptom management mainly evaluates symptoms based on their frequency, severity, and distress, and typically treats different symptoms as independent problems requiring separate interventions (9, 10). Furthermore, most previous studies on chemotherapy-related symptoms have adopted cross-sectional surveys, longitudinal follow-ups, or retrospective assessment designs (11–13). Although these studies can describe the incidence, severity, and overall trends of symptoms, they have notable limitations. On the one hand, retrospective reports are prone to recall bias, which makes it difficult to accurately capture the short-term fluctuation patterns of symptoms. On the other hand, most studies have focused on individual symptoms or overall symptom burden, with limited investigation into the time-varying interactions among symptoms. Consequently, it is difficult to determine whether changes in one symptom at a previous time point are associated with changes in other symptoms at a subsequent time point, thereby hindering a deeper understanding of the developmental processes and intrinsic relationships of chemotherapy-related symptoms.

In recent years, symptom network analysis has provided a novel methodological approach for understanding the co-occurrence and interactions of multiple symptoms in cancer patients (14–16). This approach conceptualizes symptoms as nodes and inter-symptom associations as edges, enabling the identification of prominent symptoms and connection patterns (17). However, most symptom-network studies have been cross-sectional and therefore cannot characterize within-person short-term temporal associations (18). Longitudinal studies have shown that symptom-cluster composition and trajectories can change across chemotherapy periods (19, 20). Sun et al. (21) recently used a cross-lagged panel network (CLPN) to examine symptom relationships during chemotherapy in patients with esophageal cancer. Panel-based CLPN studies estimate cross-wave associations across a limited number of discrete assessment occasions. By contrast, ecological momentary assessment (EMA) generates intensive longitudinal observations in daily life, reduces recall bias, and captures within-person fluctuations (22–24). Multilevel vector autoregressive (mlVAR) models can then distinguish short-interval lagged associations from contemporaneous residual associations (25). CLPN and EMA/mlVAR should therefore be viewed as complementary rather than directly interchangeable approaches.

Although longitudinal panel studies have examined symptom networks and symptom clusters across broader treatment phases (19–21), evidence remains limited regarding short-term symptom changes within a chemotherapy cycle, cross-cycle comparability, and within-person temporal associations among symptoms. The first chemotherapy cycle and the early phase of sequential treatment represent an important observation window. Therefore, the present study used high-frequency EMA after the first and second cycles of postoperative adjuvant chemotherapy in patients with CRC and applied mlVAR to estimate temporal and contemporaneous networks. This design was intended to characterize short-term symptom trajectories and predictive associations, rather than establish causal symptom pathways.

## 2 Participants and methods

### 2.1 Study design

This prospective observational longitudinal study used ecological momentary assessment (EMA) to repeatedly measure early symptoms in patients with colorectal cancer during the first and second cycles of postoperative adjuvant chemotherapy. Linear mixed-effects models were used to characterize symptom trajectories over time, and a multilevel vector autoregressive (mlVAR) model was used to estimate conditional predictive associations between adjacent assessment occasions and residual symptom associations within the same assessment occasion, as well as to compare network characteristics across chemotherapy cycles.

### 2.2 Participants

Convenience sampling was used to recruit patients with colorectal cancer who received their first postoperative adjuvant chemotherapy in the gastrointestinal oncology department of a tertiary Grade A hospital between August 2025 and June 2026.

#### 2.2.1 Inclusion criteria

(1) Pathologically confirmed colon or rectal cancer; (2) completion of surgical treatment with planned adjuvant chemotherapy; (3) age ≥18 years; (4) clear consciousness and adequate communication, comprehension, and questionnaire-completion abilities; (5) ability to complete electronic questionnaires using a smartphone independently or with researcher guidance; and (6) provision of informed consent and voluntary participation.

#### 2.2.2 Exclusion criteria

(1) Tumor recurrence or distant metastasis; (2) concurrent radiotherapy, immunotherapy, or other antitumor treatments that could substantially affect the symptom profile; (3) other active malignancies; (4) severe cognitive impairment, psychiatric disorders, or inability to cooperate with follow-up; and (5) severe dysfunction of vital organs such as the heart, brain, liver, or kidneys.

#### 2.2.3 Attrition and criteria for determining the primary analysis sample

During follow-up, participants were considered to have dropped out if any of the following occurred: (1) discontinuation of chemotherapy or change in the primary treatment regimen; (2) transfer to another hospital, death, or voluntary withdrawal; or (3) loss to follow-up or inability to continue EMA follow-up for other reasons.

Because this study aimed to evaluate symptom dynamics and time-lagged associations across two consecutive chemotherapy cycles, participants included in the primary analysis were required to have sufficient within-person repeated measurements to permit construction of lagged variables. Specifically, participants had to complete at least 30 valid EMA assessments across the two cycles, with at least 12 valid assessments in each cycle. Participants who did not meet these minimum valid-observation requirements were excluded from the primary analysis. The specific reasons and numbers for all dropouts and exclusions from the primary analysis were recorded.

### 2.3 Sample size estimation and evaluation of model information

This study used dynamic symptom network analysis based on intensive longitudinal data. The precision of mlVAR parameter estimates is jointly influenced by the number of participants, the number of repeated measurements within participants, and the number of network nodes, and no unified analytical formula is currently available for determining a minimum sample size based solely on the number of nodes. Therefore, the sample size was determined with reference to previous EMA and mlVAR studies, participant availability, the number of repeated measurements per participant, and network-model complexity. Thirteen core symptom nodes were planned. Each participant underwent EMA three times daily for seven consecutive days in both cycles 1 and 2, yielding 21 planned assessments per cycle and 42 repeated assessments across the two cycles. To support robust mlVAR parameter estimation, an effective sample size of 100–120 participants was targeted; allowing for approximately 20% attrition and invalid follow-up, 125–150 patients were planned for recruitment.

### 2.4 Instruments

#### 2.4.1 General information questionnaire

A researcher-designed general information questionnaire was used to collect sociodemographic characteristics, disease- and treatment-related information, and major clinical indicators. Sociodemographic variables included age, sex, living arrangement, employment status, and type of medical insurance. Disease- and treatment-related variables included cancer type, tumor location, surgical approach, stoma status, pathological type, chemotherapy regimen, and comorbidities. Major clinical indicators included nutritional risk, albumin, hemoglobin, and Nutritional Risk Screening 2002 (NRS-2002) score.

#### 2.4.2 Chemotherapy timing and symptom-management medication record form

For each chemotherapy cycle, chemotherapy start time, infusion completion time, specific chemotherapy regimen, route of administration, and medications used for symptom management were extracted from the hospital electronic medical record and nursing record systems. According to the specific drug composition, chemotherapy regimens were further categorized as oxaliplatin-containing or non-oxaliplatin regimens.

#### 2.4.3 Ecological momentary assessment items for core symptoms

Core-symptom EMA items were developed with reference to the core symptom-severity items of the Chinese Version of the M. D. Anderson Symptom Inventory (MDASI-C), supplemented by the M. D. Anderson gastrointestinal cancer symptom assessment module and the common symptom profile after chemotherapy for colorectal cancer. The MDASI-C was translated and validated by Wang et al. (26). The original instrument contains 13 symptom-severity items and 6 symptom-interference items, each scored on a 0–10 numeric rating scale, and has demonstrated good reliability and validity for multisymptom assessment in Chinese patients with cancer. Given the high-frequency repeated-measurement characteristics of EMA, 13 symptoms closely related to early symptom management after chemotherapy for colorectal cancer were selected for repeated assessment: nausea, vomiting, fatigue, loss of appetite, diarrhea, constipation, abdominal distension, abdominal pain, sleep disturbance, anxiety, depression, dry mouth, and numbness/tingling in the hands and feet. Each symptom was rated from 0 to 10, where 0 indicated ‘no symptom’ and 10 indicated ‘the worst symptom severity.’ In accordance with EMA methodology, symptom severity was assessed ‘right now’ or ‘since the last assessment’; sleep disturbance primarily referred to the most recent sleep episode or sleep-related difficulty since the previous assessment.

### 2.5 Data collection

Data were collected by combining electronic medical record extraction with a WeChat Mini Program-based EMA platform. Before the formal survey, uniformly trained researchers introduced the study purpose, survey procedures, Mini Program operation, and relevant precautions to participants. A baseline symptom assessment was completed before treatment administration in each chemotherapy cycle. Beginning after completion of chemotherapy infusion, EMA monitoring was conducted for seven consecutive days, with assessments delivered daily during three time windows: 08:00–10:00, 13:00–15:00, and 18:00–20:00. Twenty-one EMA assessments were planned per cycle and 42 across the two cycles. The EMA platform automatically recorded the actual submission time of each questionnaire. The exact number of hours from infusion completion to each assessment was calculated from the submission time and the infusion completion time, while study day and within-day assessment period were also recorded.

### 2.6 Quality control

Before study initiation, all researchers received standardized training to ensure consistency in participant recruitment, informed consent, questionnaire guidance, data extraction, and data verification. A pilot survey was conducted before formal data collection, and questionnaire delivery times, completion procedures, and item wording were optimized based on feedback. Before each observation cycle, researchers assisted participants with WeChat Mini Program registration and operational training. During data collection, the system automatically sent reminders at preset times; participants who did not complete an assessment within the designated time window received additional reminders by telephone or WeChat. Researchers monitored completion status daily through the system backend, promptly checked missing, delayed, or unusually clustered submissions, and recorded the relevant reasons. The same symptom items, assessment methods, and scoring criteria were used in cycles 1 and 2 to ensure cross-cycle comparability. All data were checked according to prespecified data-cleaning criteria, and abnormal records were processed only after verification against the original system and relevant study records.

### 2.7 Statistical analysis

Continuous variables were summarized as mean ± standard deviation or median (interquartile range), depending on their distribution, and categorical variables as frequencies and percentages. Baseline characteristics were compared between participants included in the primary analysis and those not included. Independent-samples t tests or Mann–Whitney U tests were used for continuous variables, and χ² tests or Fisher’s exact tests for categorical variables. Standardized mean differences (SMDs) were calculated to evaluate between-group balance. Linear mixed-effects models were used to analyze dynamic changes in the 13 core symptoms across the two chemotherapy cycles, with symptom score as the dependent variable; post-chemotherapy time phase, chemotherapy cycle, and their interaction as fixed effects; and participant as a random intercept. Estimated marginal means were used to describe symptom levels across time phases.

Overall completion was calculated from the 42 planned EMA assessments per participant across the two cycles, and missingness was summarized separately by chemotherapy cycle, study day, and within-day assessment period. Missing symptom scores were not multiply imputed for the primary network analysis. After lagged variables were constructed, only complete observation pairs containing information at both the current and corresponding lagged assessment were included. To assess whether missingness followed a systematic pattern, a mixed-effects logistic regression model was fitted with whether each planned EMA was missing as the dependent variable and participant as a random intercept. Predictors included overall symptom severity at the preceding EMA, chemotherapy cycle, study day, within-day assessment period, time since chemotherapy infusion completion, oxaliplatin-containing regimen, age, sex, comorbidities, and NRS-2002 score, with particular attention to whether greater symptom burden was associated with missingness at the subsequent EMA.

Dynamic symptom networks were constructed using multilevel vector autoregression (mlVAR), with all 13 symptom scores standardized before modeling. The primary analysis used a VAR (1) model in which symptom states at one adjacent within-day EMA occasion predicted those at the next adjacent occasion. Thus, only morning-to-afternoon and afternoon-to-evening within-day lag-1 relationships were modeled; the overnight interval from evening to the following morning was not treated as equivalent to a within-day lag-1 observation. ‘Participant ID–chemotherapy cycle’ was treated as an independent time-series unit, and no lagged relationship was established across the two chemotherapy cycles. Temporal networks used standardized regression coefficients to represent directed predictive associations between symptoms, whereas contemporaneous networks used residual partial correlations after controlling for lagged effects to represent conditional associations among symptoms at the same assessment occasion. Out-strength and in-strength were the primary temporal centrality measures, with net out-strength treated as exploratory. Temporal and contemporaneous networks were further estimated separately for cycles 1 and 2. Network-structure correlations and global strength were calculated, and 1,000-permutation tests were used to compare global strength and the maximum edge-weight difference between cycles; changes in major edge weights across cycles were also described.

Multiple sensitivity analyses were conducted to evaluate the effects of potential confounders and model specifications. First, separate mixed-effects models were fitted for each of the 13 symptoms, with fixed effects for time since chemotherapy infusion completion, within-day assessment period, chemotherapy cycle, oxaliplatin-containing regimen, antiemetic use, age, sex, comorbidities, and NRS-2002 score; standardized residuals were extracted and used to reconstruct the dynamic networks. Second, the network analysis was repeated after restricting the sample to patients receiving oxaliplatin-containing regimens to evaluate the influence of regimen heterogeneity. A VAR (2) model was then fitted with both t−1 and t−2 symptom information and compared with VAR (1) using, as far as possible, the same analyzable observation set. When comparable fit indices were available, Akaike information criterion (AIC) and Bayesian information criterion (BIC) were compared, together with lag-1 network structure, major temporal edges, and out-strength rankings. Additional analyses were repeated using a ±60-min valid completion window, adjustment for fixed time effects and within-day assessment period, and detrended residuals. Robustness of the primary findings was evaluated by comparing network-structure correlations, major edge weights, and out-strength rankings between each sensitivity model and the primary model.

All data management, longitudinal analyses, and dynamic symptom network modeling were performed in R (version 4.4.1), primarily using the mlVAR (version 0.5.5), lme4 (version 2.0-6), and emmeans (version 2.0.4) packages. All statistical tests were two-sided with α = 0.05. For multiple tests involving the 13 symptoms, the Benjamini–Hochberg procedure was used to control the false discovery rate (FDR).

## 3 Results

### 3.1 Participants and EMA completion

A total of 202 patients receiving their first postoperative adjuvant chemotherapy for colorectal cancer were enrolled during the study period. During follow-up, 67 patients discontinued follow-up because of voluntary withdrawal, loss to follow-up, change in the primary chemotherapy regimen, discontinuation of chemotherapy, transfer to another hospital, or death. An additional 25 patients did not enter the primary analysis because the number of valid observations did not meet the prespecified requirement. Ultimately, 110 patients were included, accounting for 54.5% of the initial sample. The detailed participant inclusion and attrition process is shown in **Figure 1** and **Supplementary Table S1A**. Baseline comparisons between included and non-included participants showed standardized differences of 0.209 for age and −0.151 for nutritional risk; the remaining comparisons are presented in **Supplementary Table S1B**.

**Figure 1:**
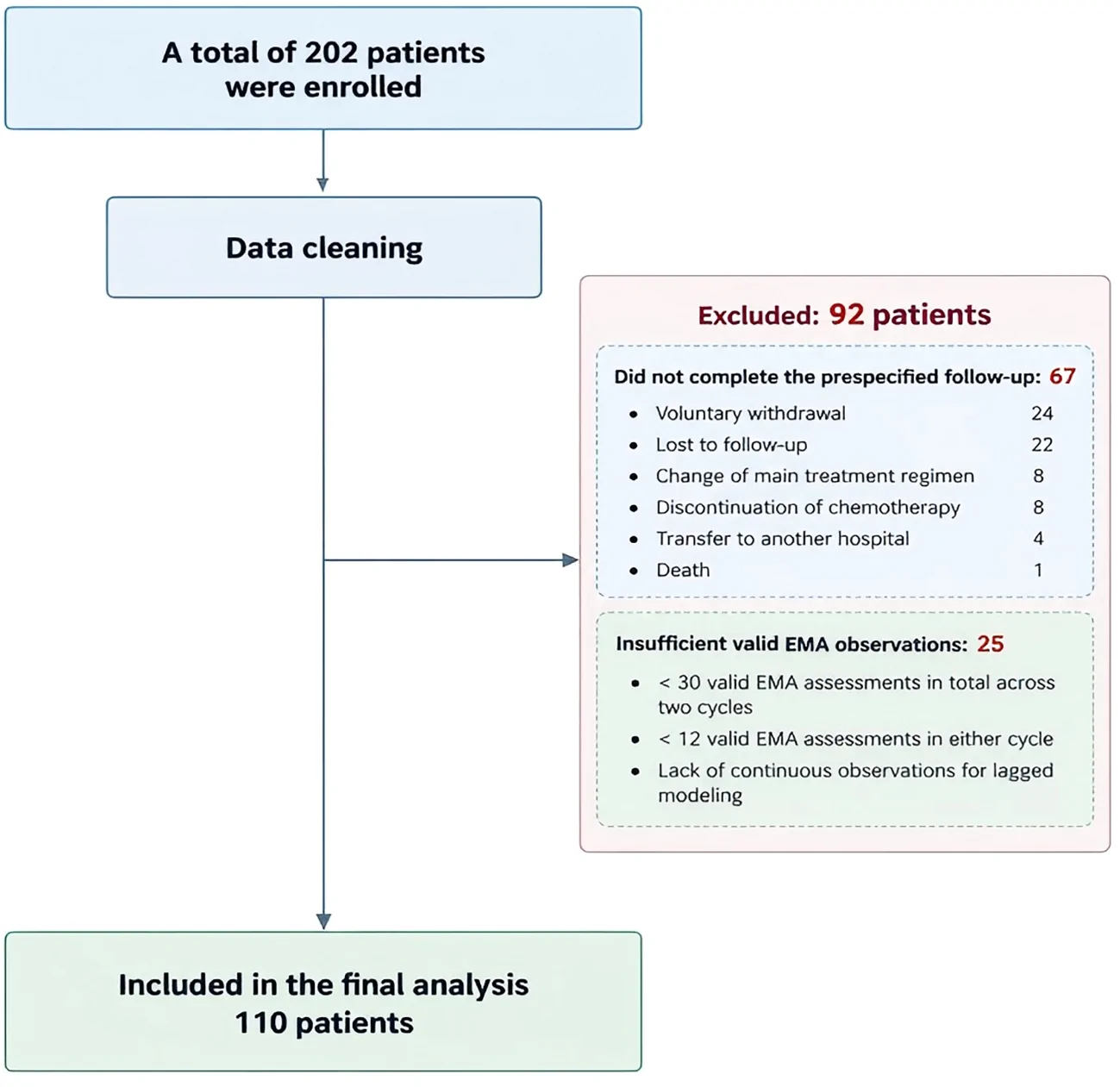
Participant selection flowchart.

The 110 patients included in the final analysis had a mean age of 61.60 ± 9.63 years; 73 (66.4%) were male, 76 (69.1%) had stage III disease, 24 (21.8%) had a stoma, and 25 (22.7%) were at nutritional risk. CAPOX and mFOLFOX6 accounted for 44.5% and 38.2%, respectively, comprising 91 patients (82.7%) in total. The main baseline characteristics are presented in **Table 1**.

**Table 1:** Baseline characteristics of participants.

Variable	Category	Mean ± SD or n (%)
Age (years)		61.60 ± 9.63
Height (cm)		165.79 ± 7.78
Weight (kg)		61.90 ± 10.04
BMI (kg/m²)		22.50 ± 3.17
Sex	Male	73 (66.4)
	Female	37 (33.6)
Living arrangement	Living with spouse	75 (68.2)
	Living with children	27 (24.5)
	Living with others	7 (6.4)
	Living alone	1 (0.9)
Employment status	Employed	20 (18.2)
	Retired	24 (21.8)
	Farmer	37 (33.6)
	Unemployed	22 (20.0)
	Other	7 (6.4)
Medical insurance	Employee medical insurance	49 (44.5)
	Urban and rural resident medical insurance	42 (38.2)
	Cross-regional medical insurance	7 (6.4)
	Self-pay	12 (10.9)
Educational level	Primary school or below	27 (24.5)
	Junior high school	49 (44.5)
	Senior high school or technical secondary school	18 (16.4)
	Junior college	7 (6.4)
	Bachelor’s degree or above	9 (8.2)
Marital status	Married	99 (90.0)
	Other	11 (10.0)
Primary caregiver	Spouse	63 (57.3)
	Children	39 (35.5)
	Other	8 (7.3)
Disease- and treatment-related characteristics		
Albumin (g/L)		40.31 ± 4.14
Hemoglobin (g/L)		120.81 ± 14.72
NRS-2002 score		1.99 ± 0.89
Diagnosis	Colon cancer	67 (60.9)
	Rectal cancer	43 (39.1)
Tumor location	Colon	35 (31.8)
	Sigmoid colon	30 (27.3)
	Rectosigmoid junction	2 (1.8)
	Rectum	43 (39.1)
Surgical approach	Laparoscopic	90 (81.8)
	Open	13 (11.8)
	Robotic	7 (6.4)
Stoma	Yes	24 (21.8)
	No	86 (78.2)
Stoma type	None	86 (78.2)
	Temporary	18 (16.4)
	Permanent	6 (5.5)
Pathological type	Colon adenocarcinoma	67 (60.9)
	Rectal adenocarcinoma	43 (39.1)
Pathological stage	Stage II	34 (30.9)
	Stage III	76 (69.1)
Cycle 1 chemotherapy regimen	CAPOX	49 (44.5)
	mFOLFOX6	42 (38.2)
	Capecitabine monotherapy	3 (2.7)
	Other	16 (14.5)
Comorbidities	None	67 (60.9)
	Hypertension	16 (14.5)
	Type 2 diabetes	7 (6.4)
	Hypertension and type 2 diabetes	17 (15.5)
	Other	3 (2.7)
Nutritional risk	Yes	25 (22.7)
	No	85 (77.3)

A total of 4,620 EMA assessments were planned across the two chemotherapy cycles, and 4,134 complete assessments were obtained, yielding an overall completion rate of 89.5%. Completion rates in cycles 1 and 2 were 90.5% and 88.5%, respectively. Completion rates across the morning, afternoon, and evening assessment periods ranged from 88.5% to 90.3%, and those across study days 1–7 ranged from 87.7% to 91.4%. No item-level symptom data were missing within completed assessments (**Supplementary Table S2**). Missingness-pattern analysis showed a slightly higher probability of missing assessments in cycle 2 than in cycle 1 (OR = 1.241, 95% CI: 1.027–1.499, P = 0.026), although this association was no longer statistically significant after BH correction (q = 0.307). No significant associations were identified for the remaining demographic, clinical, or time-related variables. The mean symptom burden at the preceding assessment was also not significantly associated with subsequent EMA missingness (OR = 0.968, 95% CI: 0.862–1.087, P = 0.583, BH q = 0.846; **Supplementary Table S3**).

### 3.2 Symptom severity and dynamic changes

Across the 4,134 complete assessments, fatigue, sleep disturbance, and loss of appetite had relatively high mean scores of 3.75 ± 1.63, 2.61 ± 1.52, and 2.41 ± 1.52, respectively. Scores for the 13 symptoms across the two cycles are shown in **Table 2**. LMM results indicated significant time effects for all 13 symptoms (FDR-corrected P ≤ 0.017), whereas none of the time × cycle interaction effects were statistically significant (FDR-corrected P = 0.761–0.976). Fatigue, loss of appetite, abdominal distension, sleep disturbance, and dry mouth generally reached higher levels during the middle post-chemotherapy period and subsequently declined; nausea and anxiety generally decreased over time, depression increased during the later period, and numbness/tingling in the hands and feet decreased during the middle period and then increased again. Symptom trajectories are shown in **Figure 2**, trajectories on a common scale in **Supplementary Figure S1**, and estimated marginal means in **Supplementary Table S4**. For the main effect of cycle, compared with cycle 1, scores in cycle 2 increased by 0.567 for fatigue, 0.338 for loss of appetite, 0.394 for sleep disturbance, 0.132 for dry mouth, and 0.504 for numbness/tingling, with all FDR-corrected P values < 0.001; anxiety decreased by 0.083 points (FDR-corrected P = 0.033). No significant between-cycle differences were observed for the remaining symptoms after multiple-comparison correction (**Table 3**; **Supplementary Table S5**).

**Table 2:** Scores of the 13 symptoms across two chemotherapy cycles.

Symptom	Cycle 1 mean ± SD	Cycle 2 mean ± SD	Overall >0, %
Nausea	1.11 ± 1.26	1.11 ± 1.24	54.5
Vomiting	0.26 ± 0.55	0.27 ± 0.55	21.9
Fatigue	3.48 ± 1.62	4.02 ± 1.60	93.3
Loss of appetite	2.25 ± 1.50	2.58 ± 1.53	82.0
Diarrhea	0.87 ± 1.01	0.90 ± 1.02	50.7
Constipation	1.01 ± 1.05	1.06 ± 1.06	58.1
Abdominal distension	1.29 ± 1.11	1.34 ± 1.13	68.7
Abdominal pain	1.13 ± 1.09	1.14 ± 1.10	62.7
Sleep disturbance	2.42 ± 1.50	2.80 ± 1.51	85.0
Anxiety	1.45 ± 1.25	1.37 ± 1.29	66.4
Depression	1.13 ± 1.14	1.17 ± 1.14	60.8
Dry mouth	1.22 ± 1.08	1.35 ± 1.10	68.1
Numbness/tingling	0.79 ± 0.95	1.30 ± 1.12	58.9

**Figure 2:**
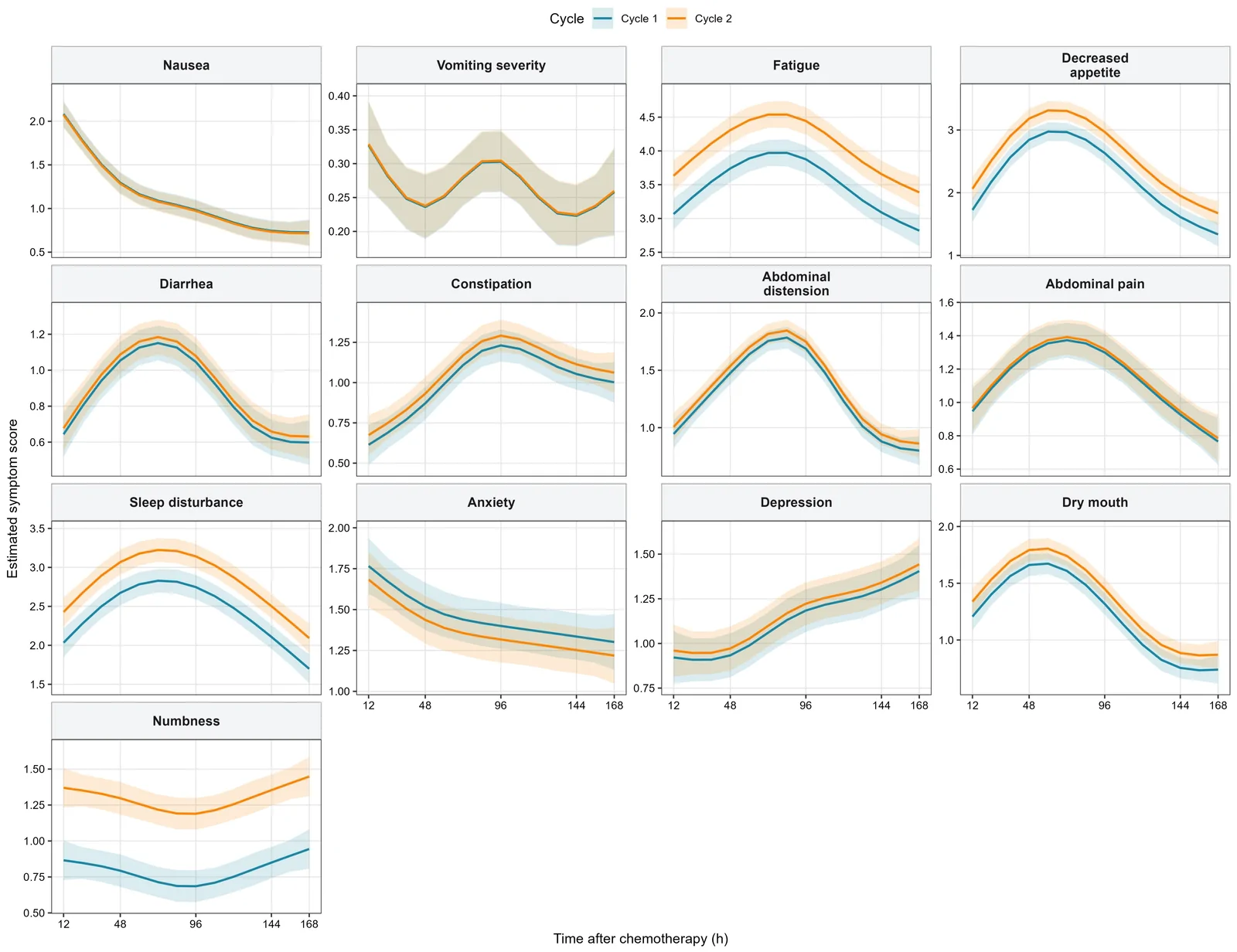
Dynamic trajectories of the 13 symptoms across two chemotherapy cycles.

**Table 3:** Time effects, cycle effects, and time × cycle interaction effects for the 13 symptoms.

Symptom	Time F	Time FDR-adjusted P	Cycle F	Cycle FDR-adjusted P	Interaction F	Interaction FDR-adjusted P
Nausea	108.49	<0.001	0.10	0.817	1.38	0.761
Vomiting	3.03	0.017	0.01	0.922	0.50	0.869
Fatigue	59.38	<0.001	127.31	<0.001	0.28	0.968
Loss of appetite	149.73	<0.001	54.61	<0.001	1.43	0.761
Diarrhea	51.01	<0.001	1.38	0.311	0.12	0.976
Constipation	38.94	<0.001	3.98	0.085	0.67	0.802
Abdominal distension	117.24	<0.001	3.76	0.085	1.14	0.761
Abdominal pain	30.96	<0.001	0.29	0.699	1.11	0.761
Sleep disturbance	73.40	<0.001	94.18	<0.001	0.89	0.761
Anxiety	10.14	<0.001	5.92	0.033	0.96	0.761
Depression	22.95	<0.001	1.49	0.311	1.10	0.761
Dry mouth	130.84	<0.001	18.76	<0.001	0.66	0.802
Numbness/tingling	5.98	<0.001	251.37	<0.001	0.92	0.761

P values are FDR-adjusted P values using the Benjamini–Hochberg procedure.

### 3.3 Overall dynamic symptom network characteristics

In the overall temporal network, 30 of the 156 cross-symptom predictive edges remained statistically significant after BH correction (q < 0.05). The strongest positive cross-symptom temporal associations were observed from loss of appetite to subsequent fatigue (β = 0.123), abdominal distension (β = 0.110), and sleep disturbance (β = 0.105); from sleep disturbance to subsequent fatigue (β = 0.102); and from nausea to subsequent loss of appetite (β = 0.100). Associations from abdominal distension to subsequent loss of appetite and from dry mouth to subsequent diarrhea, loss of appetite, and abdominal distension were also comparatively prominent. The patient-level bootstrap 95% CIs for these major edges did not include zero, indicating greater precision regarding the direction of these individual edge estimates; this should not be interpreted as evidence of overall network stability. The overall temporal predictive network is shown in **Figure 3**, the major temporal associations in **Table 4A**, the complete results in **Supplementary Table S6**, and the bootstrap intervals for the major edges in **Supplementary Figure S2**. In the overall contemporaneous network, the strongest positive conditional associations were observed between fatigue and sleep disturbance (r = 0.144), abdominal distension and abdominal pain (r = 0.144), anxiety and depression (r = 0.135), loss of appetite and dry mouth (r = 0.107), and fatigue and loss of appetite (r = 0.103). The overall contemporaneous network is shown in **Figure 4**, the major contemporaneous associations in **Table 4B**, and the complete results in **Supplementary Table S7**.

**Figure 3:**
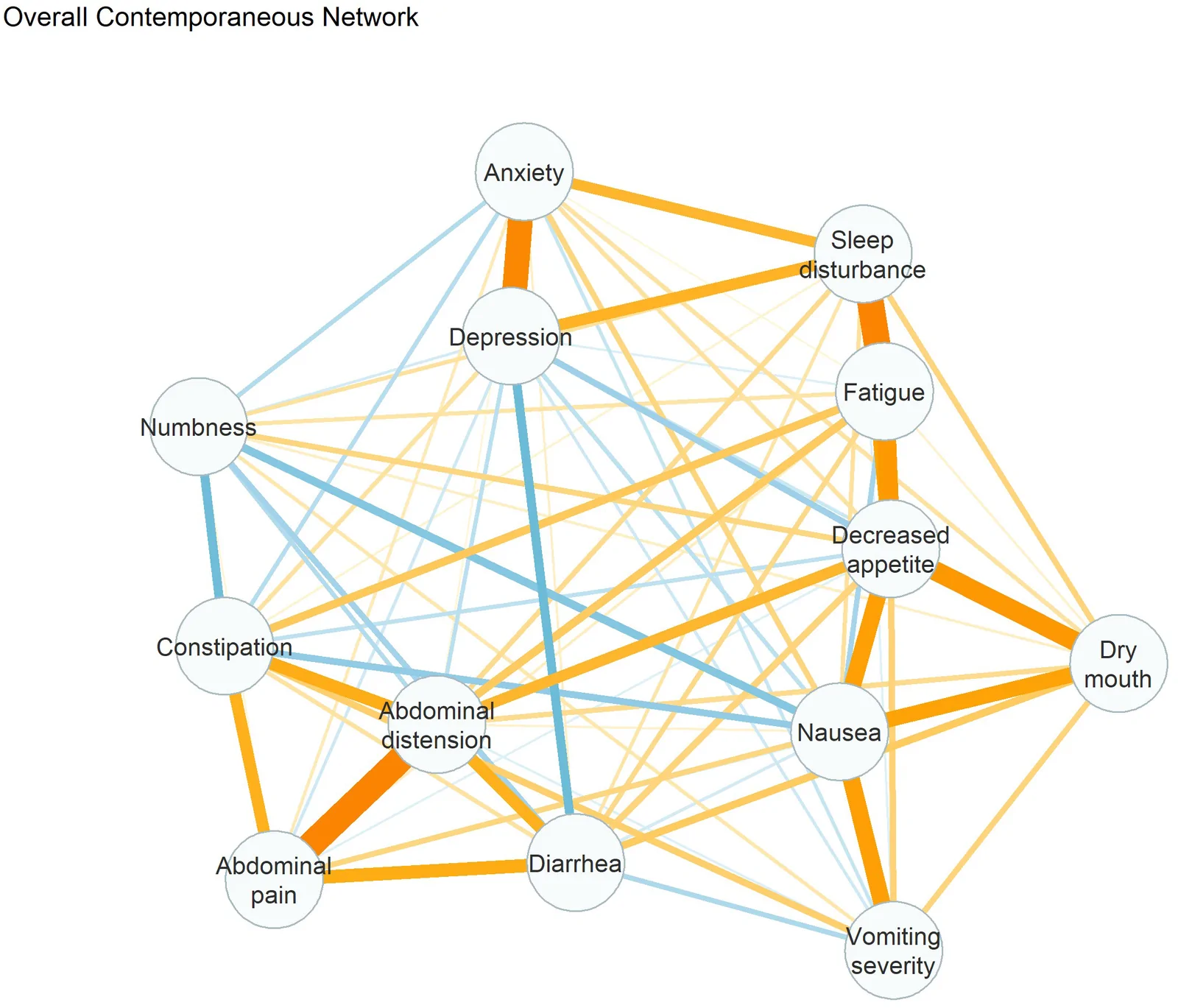
Overall temporal symptom network structure. Arrows represent predictive associations from symptoms at the previous assessment to symptoms at the subsequent assessment; self-loops represent autoregressive effects. Orange indicates positive associations, blue indicates negative associations, and line width corresponds to the absolute magnitude of the edge weight.

**Table 4A:** Major cross-symptom temporal predictive associations.

Temporal association	β	95% Wald CI	95% Bootstrap CI	BH q
Loss of appetite→Fatigue	0.123	0.093–0.153	0.097–0.147	<0.001
Loss of appetite→Abdominal distension	0.110	0.070–0.150	0.068–0.144	<0.001
Loss of appetite→Sleep disturbance	0.105	0.072–0.139	0.078–0.137	<0.001
Sleep disturbance→Fatigue	0.102	0.071–0.133	0.073–0.130	<0.001
Nausea→Loss of appetite	0.100	0.069–0.132	0.075–0.124	<0.001
Abdominal distension→Loss of appetite	0.087	0.054–0.120	0.051–0.120	<0.001
Dry mouth→Diarrhea	0.085	0.050–0.119	0.054–0.111	<0.001
Dry mouth→Loss of appetite	0.083	0.051–0.114	0.055–0.112	<0.001
Dry mouth→Abdominal distension	0.082	0.048–0.116	0.052–0.115	<0.001
Loss of appetite→Abdominal pain	0.078	0.043–0.113	0.048–0.110	<0.001

β denotes the standardized temporal regression coefficient; Bootstrap CI denotes the 95% percentile interval obtained by patient-level cluster resampling; BH q denotes the Benjamini–Hochberg-adjusted result across the 156 cross-symptom edges.

**Table 4B:** Major contemporaneous conditional associations.

Contemporaneous association	Partial r	95% Bootstrap CI
Fatigue—Sleep disturbance	0.144	0.107–0.176
Abdominal distension—Abdominal pain	0.144	0.110–0.170
Anxiety—Depression	0.135	0.093–0.174
Loss of appetite—Dry mouth	0.107	0.082–0.134
Fatigue—Loss of appetite	0.103	0.063–0.133
Nausea—Vomiting	0.093	0.064–0.127
Nausea—Loss of appetite	0.092	0.061–0.126
Nausea—Dry mouth	0.082	0.046–0.117
Loss of appetite—Sleep disturbance	0.081	0.047–0.110
Diarrhea—Abdominal pain	0.068	0.027–0.103

r denotes the residual partial correlation coefficient after controlling for lagged effects and the other symptoms; Bootstrap CI denotes the 95% percentile interval obtained by patient-level cluster resampling.

**Figure 4:**
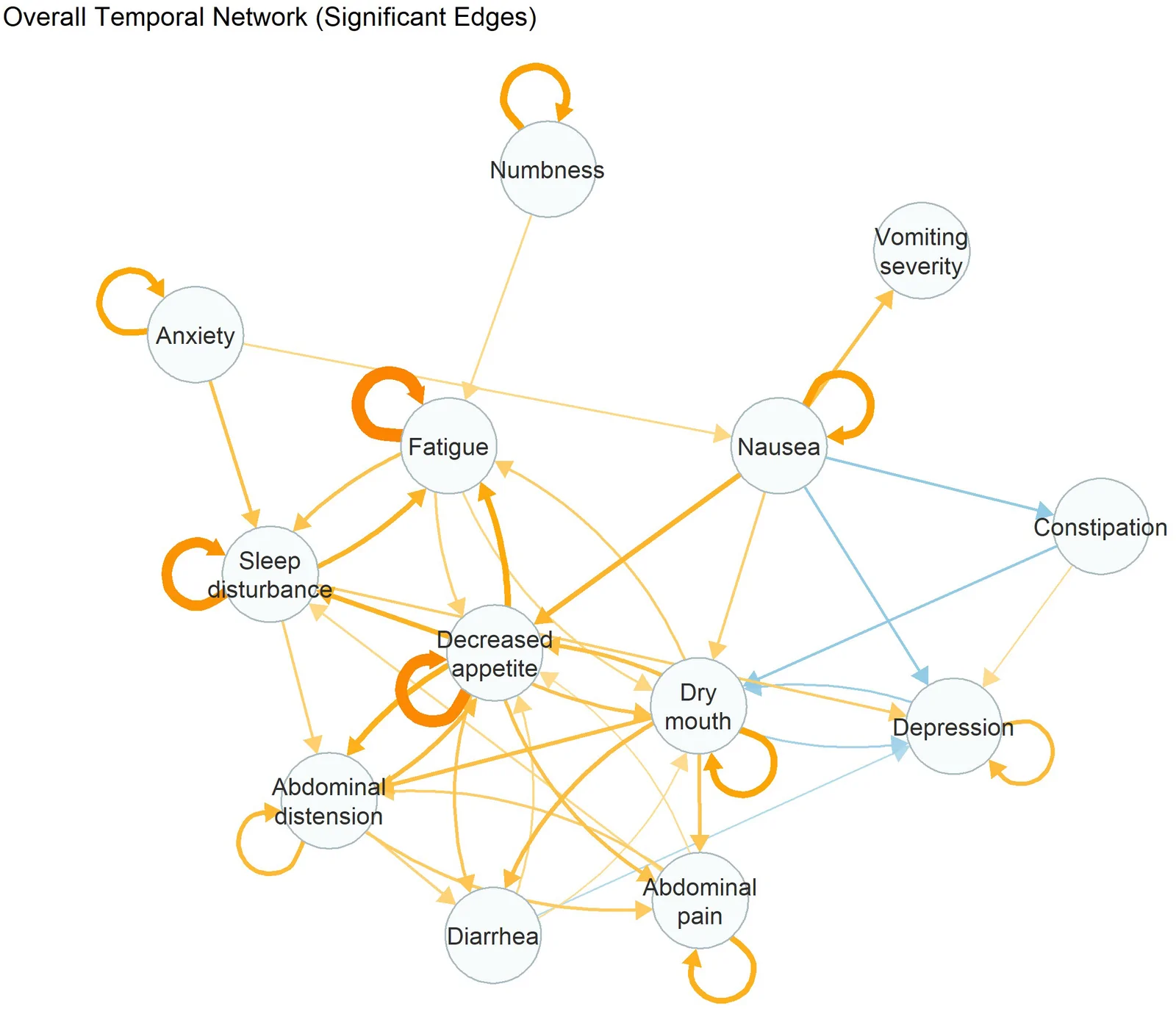
Overall contemporaneous symptom network structure. Edges represent residual conditional associations after controlling for lagged effects and the other symptoms. Orange indicates positive associations, blue indicates negative associations, and line width corresponds to the absolute magnitude of the edge weight.

### 3.4 Centrality and centrality stability

In the primary temporal network, loss of appetite had the highest out-strength (0.629), followed by dry mouth (0.491) and nausea (0.457). Symptoms with higher contemporaneous strength were loss of appetite (0.584), abdominal distension (0.460), and nausea (0.457). Complete centrality indices are presented in **Table 5**, and out-strength comparisons are shown in **Figure 5**.

**Table 5:** Centrality indices of the overall symptom network.

Symptom	Out-strength	In-strength	Contemporaneous strength
Loss of appetite	0.629	0.510	0.584
Dry mouth	0.491	0.446	0.345
Nausea	0.457	0.211	0.457
Sleep disturbance	0.365	0.439	0.449
Abdominal distension	0.341	0.423	0.460
Fatigue	0.333	0.474	0.402
Abdominal pain	0.251	0.333	0.341
Diarrhea	0.242	0.321	0.356
Depression	0.237	0.305	0.365
Anxiety	0.231	0.091	0.319
Constipation	0.212	0.220	0.345
Vomiting	0.178	0.232	0.249
Numbness/tingling	0.171	0.133	0.234

**Figure 5:**
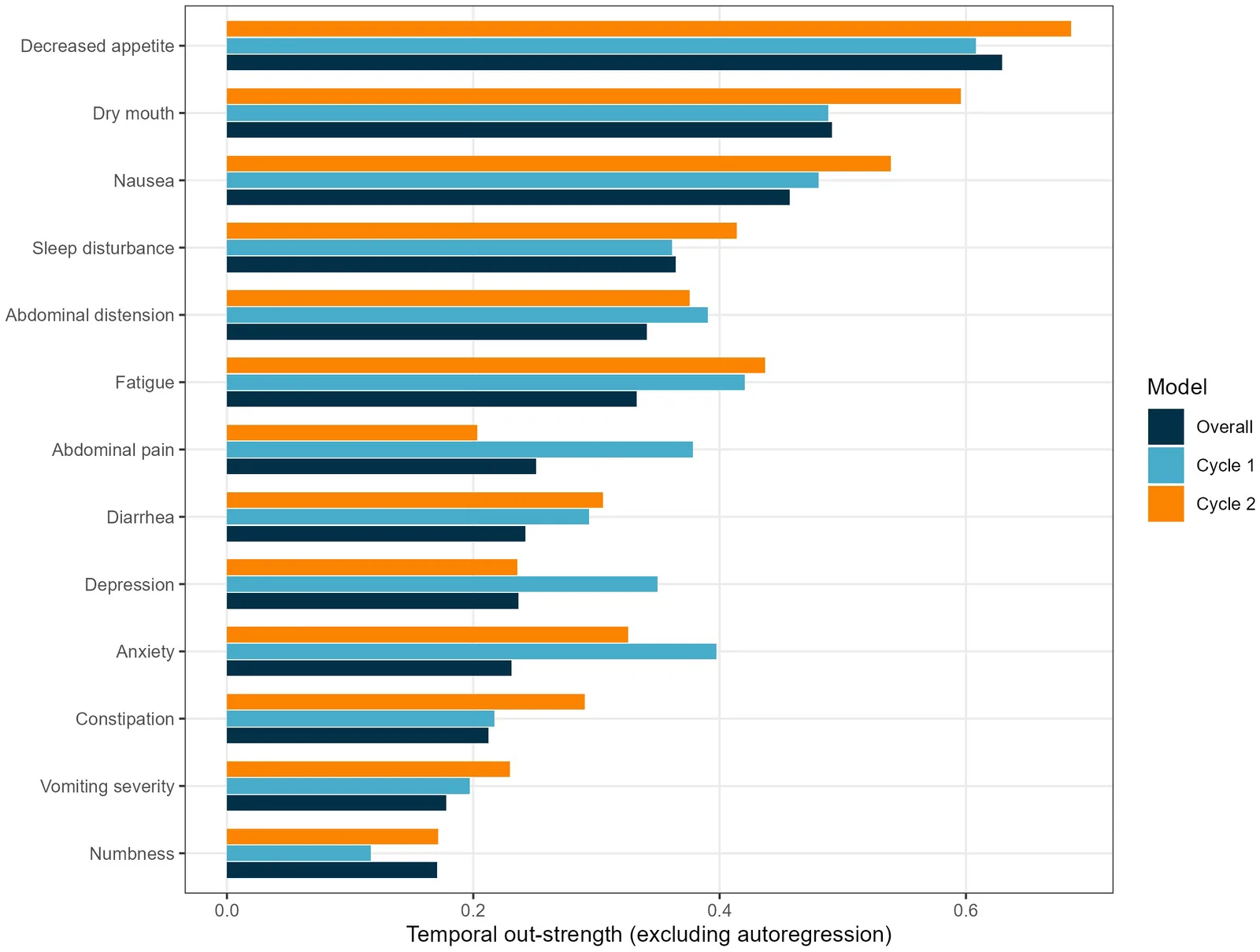
Temporal out-strength of symptoms in the overall network and the two chemotherapy cycles. Out-strength is the sum of the absolute values of all cross-symptom outgoing edges for the corresponding symptom, excluding autoregressive effects.

Patient-level bootstrap analysis showed that loss of appetite ranked first in out-strength in 95.5% of resamples, with a 95% percentile rank range of 1–2. In the case-dropping analysis, after 50% of participants were randomly removed, the 5th percentiles of the Pearson correlations with the full-sample centrality estimates were 0.857 for temporal out-strength, 0.869 for temporal in-strength, and 0.757 for contemporaneous strength; the corresponding 5th percentiles of the Spearman rank correlations were 0.735, 0.846, and 0.631, respectively. These findings indicate comparatively high stability of the temporal centrality estimates across the examined case-dropping proportions, whereas contemporaneous strength rankings were somewhat less stable (**Supplementary Table S8**; **Supplementary Figure S3**).

### 3.5 Comparison of networks between the two chemotherapy cycles

The global strengths of the temporal networks were 4.699 in cycle 1 and 4.808 in cycle 2, whereas those of the contemporaneous networks were 2.737 and 2.533, respectively. Pearson correlations of edge weights between cycles were 0.663 for the temporal networks and 0.697 for the contemporaneous networks, suggesting broad structural comparability alongside changes in some local associations. Cycle-specific networks are shown in **Figures 6**, **7**, overall indices in **Supplementary Table S9**, local associations with larger changes in **Supplementary Table S10**, and difference heatmaps in **Supplementary Figures S4**, **S5**. Because the local edge comparisons were primarily descriptive, differences in individual point estimates should not be interpreted as statistically significant between-cycle differences in the absence of corresponding inferential tests.

**Figure 6:**
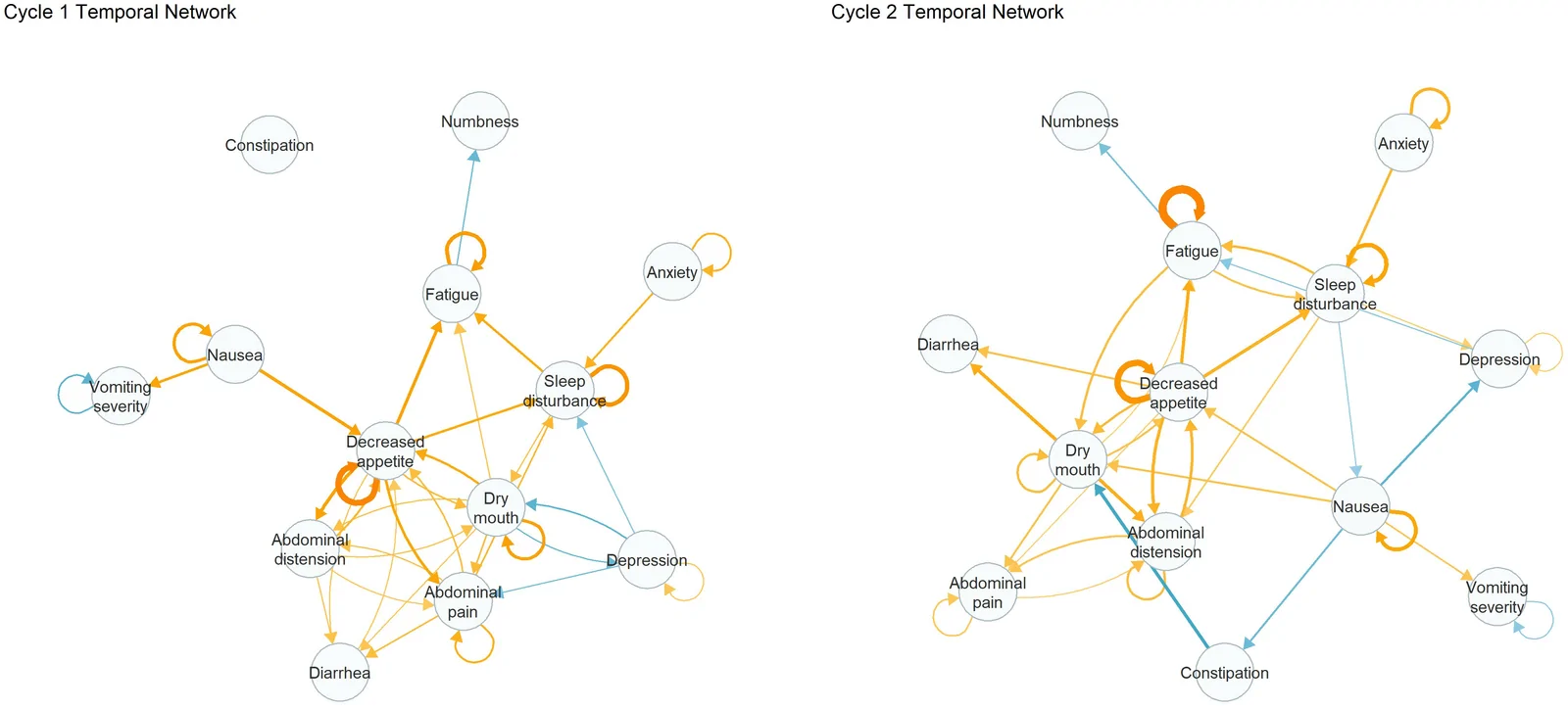
Temporal symptom networks in cycles 1 and 2.

**Figure 7:**
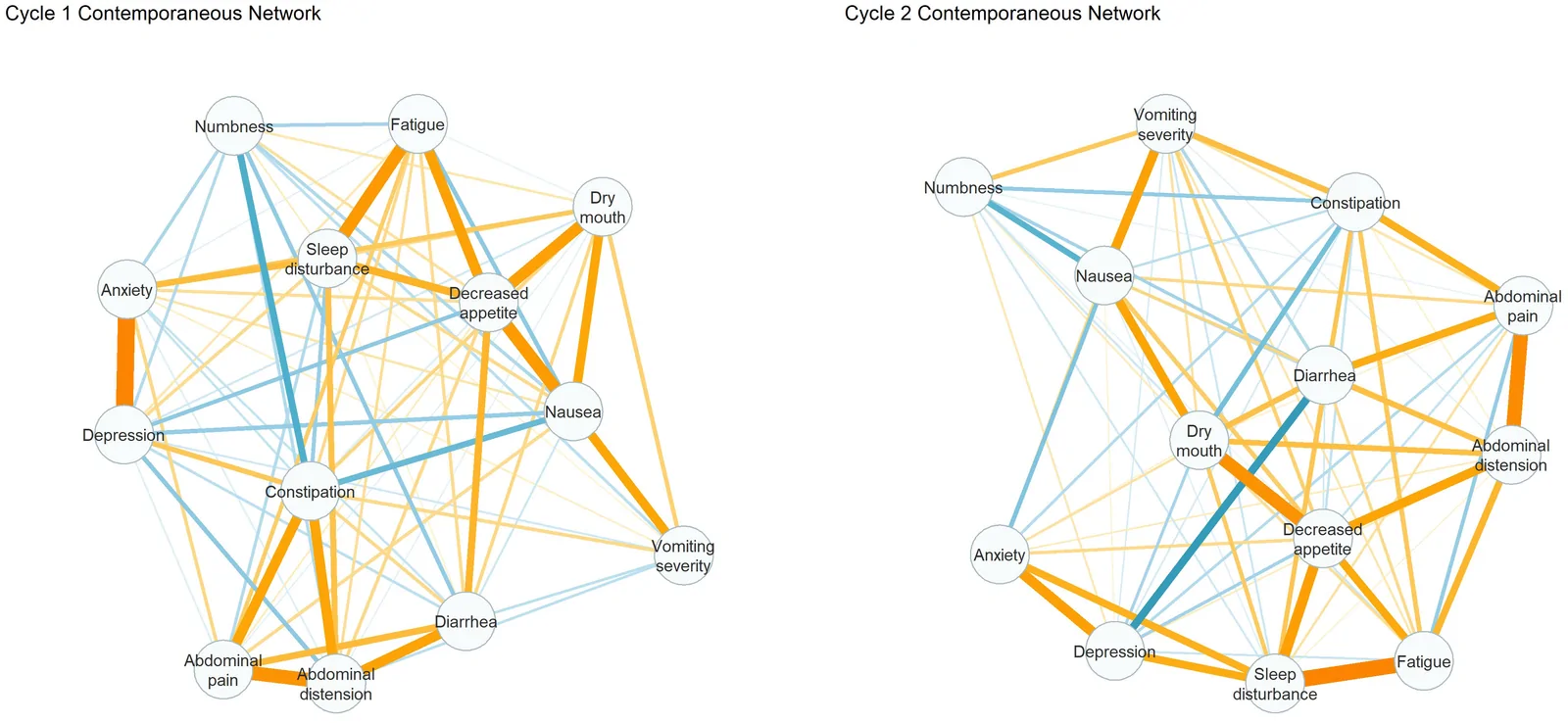
Contemporaneous symptom networks in cycles 1 and 2.

### 3.6 Covariate adjustment and sensitivity analyses

After further adjustment for nonlinear post-chemotherapy time and its interaction with cycle, time of day, supportive medications, age, sex, BMI, comorbidities, NRS-2002 score, pathological stage, and chemotherapy regimen, the structural correlations of the temporal and contemporaneous networks with the primary model were 0.700 and 0.896, respectively. After adjustment, the positive association between loss of appetite and fatigue remained (β = 0.077, 95% CI: 0.044–0.111, BH q < 0.001), whereas the edges from loss of appetite to abdominal distension, dry mouth to loss of appetite, and dry mouth to abdominal distension were substantially attenuated. The out-strength ranking also changed: nausea ranked highest (0.343), followed by loss of appetite (0.241), sleep disturbance (0.240), and dry mouth (0.239). Major temporal associations are presented in **Supplementary Table S11A**, and the adjusted networks and out-strength comparisons are shown in **Supplementary Figures S6**, **S7**.

In a restricted analysis including only patients receiving CAPOX or mFOLFOX6, 91 patients and 2,916 valid lagged pairs were available. Structural correlations of the temporal and contemporaneous networks with the full-sample primary networks were 0.976 and 0.982, respectively, and the top three symptoms by out-strength remained loss of appetite, dry mouth, and nausea. When only 2,452 lagged pairs with a planned within-day interval of approximately 6 h were retained, structural correlations with the primary model were 0.937 for the temporal network and 0.953 for the contemporaneous network; the principal out-strength ranking and key temporal associations were generally maintained. Results are presented in **Supplementary Tables S11A, B**.

VAR(1) and VAR(2) were compared using 3,021 records with complete data at t, t−1, and t−2. VAR(2) yielded lower AIC values for fatigue, loss of appetite, diarrhea, constipation, abdominal distension, sleep disturbance, and dry mouth, with BH-adjusted q < 0.05 for the likelihood-ratio tests. However, BIC favored the more parsimonious VAR(1) model for all 13 symptoms. Model-fit comparisons are presented in **Supplementary Table S12A** and **Supplementary Figure S8**.

Other sensitivity analyses showed that restricting the completion window to ±60 min, detrending, removing temporal random slopes, and deleting the vomiting node all produced network structures that remained highly correlated with the primary model. Networks before and after detrending are shown in **Supplementary Figures S9**, **S10**. Each node equation in the primary temporal model used 3,528 valid lagged observations and estimated 26 fixed-effect coefficients, corresponding to an observation-to-fixed-effect-coefficient ratio of 135.7; when random effects and the residual scale parameter were also counted, the ratio was 86.0. Optimization was successfully completed for all node equations, but boundary singular fits occurred in all random-effects structures, indicating that complex random-effects specifications and limited within-person observations may affect the precision of some parameter estimates. Accordingly, centrality and individual-difference results should be interpreted cautiously. Detailed results are presented in **Table 6**, **Supplementary Tables S11B**, **S12B**.

**Table 6:** Network comparisons between the primary model and adjusted/sensitivity models.

Analysis	Patients, n	Lagged Pairs, n	Temporal r	Contemporaneous r	Temporal strength	Contemporaneous strength
Time- and Supportive Medication-Adjusted Residuals	110	3354	0.6945	0.8957	2.590	1.912
Detrended Residuals	110	3354	0.7129	0.8947	2.598	1.951
± 60-min Window Only	110	3096	0.9833	0.9903	4.258	2.465
Temporal Random Slopes Removed	110	3528	0.9991	0.9991	4.113	2.466
Vomiting Node Removed	110	3528	0.9997	0.9996	3.716	2.205
Extended Clinical Covariate-Adjusted Residuals	110	3354	0.7002	0.8955	2.588	1.959
Restricted to CAPOX & mFOLFOX6	91	2916	0.9765	0.9822	4.504	2.526
Planned Within-Day 6-h Intervals Only	110	2452	0.9367	0.9527	4.450	2.605

r denotes the Pearson correlation coefficient between the corresponding edge vector of each model and that of the primary network; strength is calculated from the complete set of estimated edge weights.

## 4 Discussion

This EMA study obtained intensive repeated symptom measurements after the first and second cycles of postoperative adjuvant chemotherapy in patients with CRC. Gastrointestinal symptoms, fatigue, and sleep disturbance showed marked short-term fluctuations, whereas numbness/tingling and depression tended to increase later in the observation period. Loss of appetite, dry mouth, and nausea showed comparatively prominent outgoing temporal associations and preceded changes in several other symptoms within the observed assessment intervals. Although the relative out-strength ranking changed after multivariable adjustment, these three symptoms remained among the more prominent symptoms across the primary and sensitivity analyses. These findings represent temporal predictive associations and do not establish causal relationships between symptoms. The estimated network structures were broadly comparable across the first two chemotherapy cycles, supporting further investigation of phase-sensitive, longitudinal symptom monitoring.

### 4.1 Early post-chemotherapy symptoms in CRC patients exhibit pronounced temporally dynamic changes

The early post-chemotherapy symptom burden in patients with CRC was not static but changed substantially over time. Fatigue, loss of appetite, diarrhea, constipation, abdominal distension, abdominal pain, and sleep disturbance showed distinct temporal patterns, broadly consistent with previous reports. Earlier studies have shown that patients with CRC commonly experience nausea, fatigue, loss of appetite, and sleep disturbance during the early post-chemotherapy period (27, 28). Using repeated measurements within a chemotherapy cycle, Röhrl et al. (27) reported that symptoms such as nausea and fatigue worsened during the days after chemotherapy and subsequently improved before the next cycle, suggesting a cyclical pattern of symptom burden. By applying high-frequency EMA, the present study further characterized short-term changes in symptoms during the early post-chemotherapy period. Dry mouth tended to peak earlier, whereas numbness/tingling in the hands and feet and depression increased during the later observation period. In addition, fatigue, loss of appetite, sleep disturbance, dry mouth, and numbness/tingling were more severe in cycle 2, whereas anxiety was slightly lower, suggesting that symptom severity may evolve across repeated treatment even when the overall shape of the short-term trajectories remains broadly similar. Potential explanations include chemotherapy- and supportive-medication-related xerostomia and reduced oral intake (29–31), cumulative neurotoxicity contributing to numbness/tingling (32), and the possible influence of persistent symptom burden and treatment uncertainty on depressive symptoms (33). These mechanisms were not directly examined in the present study; nevertheless, the observed temporal heterogeneity supports phase-sensitive symptom monitoring.

### 4.2 Loss of appetite, dry mouth, and nausea show relatively prominent outgoing temporal associations

Loss of appetite, dry mouth, and nausea showed comparatively high out-strength, and loss of appetite exhibited outgoing temporal associations in both the overall and cycle-specific networks. However, after adjustment for post-chemotherapy time, time of day, supportive medications, demographic and clinical factors, and chemotherapy regimen, nausea ranked highest in out-strength, followed by loss of appetite, sleep disturbance, and dry mouth; several individual edges involving loss of appetite and dry mouth were also attenuated. This indicates that the exact ranking and magnitude of individual temporal associations are partly sensitive to covariate adjustment. Shang et al. (34) applied CLPN in perioperative patients with CRC, whereas Sun et al. (21) recently used CLPN to examine symptom dynamics during chemotherapy in patients with esophageal cancer. These panel-based approaches estimate cross-wave associations across discrete assessment occasions, whereas cross-sectional and contemporaneous network analyses characterize associations within the same assessment occasion (35, 36). By contrast, the present EMA/mlVAR analysis focused on short-interval within-person temporal associations while separately estimating contemporaneous residual associations. Higher loss-of-appetite scores temporally predicted subsequent changes in fatigue, abdominal distension, abdominal pain, and sleep disturbance, whereas nausea and dry mouth temporally predicted subsequent changes in loss of appetite. The persistence of the loss of appetite-to-fatigue association after covariate adjustment, together with the high structural correlations observed in the CAPOX/mFOLFOX6-restricted analysis, supports the robustness of the broader pattern, although not every individual edge was equally robust. Nutritional, gastrointestinal, and psychological mechanisms may contribute to these associations (37–39), but they were not directly tested. Accordingly, loss of appetite, dry mouth, and nausea should be regarded as candidate symptoms for enhanced monitoring and future hypothesis-driven research rather than as established causal intervention targets.

### 4.3 Temporal symptom associations support linked monitoring of gastrointestinal symptoms, sleep disturbance, and fatigue

The temporal network identified a sequence of predictive associations in which nausea and dry mouth preceded changes in loss of appetite, loss of appetite preceded changes in fatigue and sleep disturbance, and sleep disturbance preceded changes in fatigue. These findings support linked monitoring of gastrointestinal symptoms, appetite, sleep, and fatigue and are consistent with previous evidence regarding sleep–fatigue relationships and symptom clusters in gastrointestinal cancer (40, 41). Impaired oral intake, nutritional changes, and sleep-related recovery processes may contribute to these associations (35, 38, 42), although these mechanisms were not directly assessed. Importantly, the observed temporal associations do not demonstrate symptom propagation, triggering, or causal mediation. Unmeasured time-varying factors, including medication exposure, oral intake, hydration, daily activity, and treatment toxicity, may have influenced multiple symptoms simultaneously. Moreover, the strongest cross-symptom temporal edge was approximately 0.12, indicating a modest standardized association. Accordingly, the non-zero bootstrap intervals for major edges should be interpreted as evidence of greater precision in the direction of those individual estimates rather than as evidence of overall network stability. Centrality stability was evaluated separately using patient-level case-dropping analyses and was comparatively high for temporal out-strength and in-strength, but somewhat lower for contemporaneous strength. Centrality rankings should therefore be interpreted as measures of relative prominence within the observed 13-symptom network rather than as direct indicators of clinical effect size or intervention efficacy.

### 4.4 Symptom network structures were broadly comparable across the first two chemotherapy cycles

The temporal and contemporaneous networks estimated for cycles 1 and 2 showed moderate-to-high structural correlations, and permutation tests did not identify statistically significant between-cycle differences in global strength or maximum edge weight. These findings suggest broad comparability of the estimated network structures under the study conditions but do not demonstrate that the networks were identical. Some local edge estimates differed between cycles, and uncertainty remained for individual associations. The sensitivity analyses further supported the general robustness of the principal network pattern. Restricting the analysis to patients receiving CAPOX or mFOLFOX6 and to lagged pairs with an approximately 6-h planned interval yielded high structural correlations with the primary networks. Although VAR (2) provided a better AIC fit for several symptoms, BIC favored the more parsimonious VAR(1) specification for all 13 symptoms. Thus, the primary VAR(1) model is a reasonable representation of the prespecified within-day assessment structure, while the possibility of symptom dynamics operating over additional time scales should not be excluded. Thus, findings from the first cycle may help generate hypotheses for monitoring during subsequent cycles, but their predictive and clinical utility requires prospective validation.

### 4.5 Implications for clinical nursing practice

The findings support continuous, phase-sensitive symptom monitoring rather than reliance on isolated assessments. EMA or electronic patient-reported outcome platforms may help nursing staff identify short-term changes in gastrointestinal symptoms, fatigue, sleep disturbance, and progressively worsening numbness/tingling. Loss of appetite, dry mouth, and nausea may warrant additional attention because of their comparatively prominent outgoing temporal associations. However, because the out-strength ranking changed after covariate adjustment and the strongest temporal associations were modest in magnitude, network centrality should not be used in isolation to determine clinical priorities. Instead, network information may complement symptom severity, trajectory, treatment regimen, nutritional status, and individual patient needs when planning nursing assessment and follow-up. However, the present observational study does not demonstrate that intervening on these symptoms will reduce subsequent symptom burden. The clinical value of network-informed monitoring and linked symptom-management strategies should therefore be evaluated in prospective interventional studies.

This study has several limitations. First, participants were recruited from a single tertiary Grade A hospital, which may limit the generalizability of the findings. Second, 92 of the 202 enrolled participants were not included in the primary analysis because of voluntary withdrawal, loss to follow-up, change in the primary chemotherapy regimen, discontinuation of chemotherapy, transfer to another hospital, death, or insufficient valid EMA assessments. Baseline characteristics were compared between the 110 included and 92 non-included participants; the largest standardized difference was observed for age (SMD = 0.209), followed by nutritional risk (SMD = −0.151), whereas the remaining differences were generally small. Nevertheless, selection bias cannot be excluded because retention in an intensive EMA study may be associated with unmeasured characteristics such as digital literacy, health status, or adherence. Third, the sample-size target was pragmatic and literature-informed rather than derived from a formal simulation-based power analysis for a 13-node mlVAR model. Although the primary temporal model included 3,528 valid lagged observations and the study obtained 4,134 completed EMA assessments overall, some temporal edges were modest in magnitude and individual parameter estimates may remain imprecise. In addition, boundary singular fits occurred in the random-effects structures, indicating that the complexity of the random-effects specification relative to the available within-person observations may have affected the precision of some individual-difference estimates. Fourth, the three-times-daily EMA schedule may not have captured symptom dynamics occurring over shorter time scales, and the estimated lagged associations should therefore be interpreted within the specific assessment intervals used in this study. Although the primary analysis was restricted to within-day transitions and VAR(2) sensitivity analyses were conducted, these analyses do not fully characterize symptom dynamics occurring on substantially shorter or longer continuous time scales. Fifth, although chemotherapy regimen was included in the covariate-adjusted analyses and the major network structure was preserved in the CAPOX/mFOLFOX6 subgroup, residual heterogeneity related to regimen-specific toxicity profiles cannot be completely excluded. Sixth, only the first two chemotherapy cycles and 13 prespecified symptoms were assessed; the findings may therefore not capture symptom dynamics during later treatment or symptoms outside the measured set. Seventh, temporal prediction in an observational mlVAR model does not establish causality, and residual confounding by unmeasured time-varying factors remains possible. Finally, comparatively prominent outgoing associations do not demonstrate that targeting loss of appetite, dry mouth, or nausea will reduce subsequent symptom burden; this hypothesis requires prospective experimental evaluation.

## 5 Conclusion

Early symptoms after postoperative adjuvant chemotherapy for CRC showed marked temporal variation, and the estimated symptom-network structures were broadly comparable across the first two chemotherapy cycles. Loss of appetite, dry mouth, and nausea showed comparatively prominent outgoing temporal associations and may be candidates for enhanced monitoring and future hypothesis-driven intervention research. The broader pattern involving these symptoms was generally supported by sensitivity analyses, although covariate adjustment altered the relative out-strength ranking and attenuated some individual edges. However, the modest magnitude of individual temporal associations, uncertainty in some parameter estimates, potential selection bias, and the observational design should be considered when interpreting these findings. Causal relationships and intervention effects require confirmation in prospective experimental studies.

## Data Availability

The original contributions presented in the study are included in the article/**Supplementary Material**. Further inquiries can be directed to the corresponding author.
