# Supplementary material for: A study on the temporal network characteristics of early post-chemotherapy core symptoms in colorectal cancer patients based on ecological momentary assessment
Source: Front Oncol. 2026 Oct 27;16:1942076. doi: 10.3389/fonc.2026.1942076 (PMC13747801; doi:10.3389/fonc.2026.1942076)
Supplement: Supplementary file 1 [file DataSheet1.pdf]

Contents

Supplementary tables
Table S1 Reasons for exclusion from the analysis and baseline comparisons between included and excluded participants........ 2
Table S2 EMA completion and missing assessments........ 4
Table S3 Mixed-effects logistic regression analyses of missing EMA assessments........ 5
Table S4 Estimated marginal means for 13 symptoms across time intervals in two chemotherapy cycles........ 6
Table S5 Estimated marginal symptom means and differences between two chemotherapy cycles 8
Table S6 All 156 cross-symptom predictive coefficients in the primary temporal network........ 9
Table S7 All 78 pairwise conditional associations in the primary contemporaneous network....... 19
Table S8 Bootstrap centrality and case-dropping stability analyses........ 22
Table S9 Overall dynamic network metrics for two chemotherapy cycles........ 24
Table S10 Temporal and contemporaneous associations with the largest local edge-weight changes between two chemotherapy cycles........ 25
Table S11 Clinical covariate adjustment and sensitivity analyses........ 26
Table S12 VAR(1) versus VAR(2) comparisons and a summary of mlVAR model size........ 28

Supplementary figures
Figure S1 Trajectories of 13 symptoms on a common scale........ 30
Figure S2 Patient-level bootstrap intervals for key network edges........ 31
Figure S3 Centrality correlations after case dropping........ 32
Figure S4 Heatmap of between-cycle differences in temporal edge weights........ 33
Figure S5 Heatmap of between-cycle differences in contemporaneous edge weights........ 34
Figure S6 Networks after expanded clinical covariate residual adjustment........ 35
Figure S7 Temporal out-strength across analyses........ 36
Figure S8 AIC and BIC comparisons of models with different lag orders........ 37
Figure S9 Overall and detrended temporal networks........ 38
Figure S10 Overall and detrended contemporaneous networks........ 39

Table S1 Reasons for exclusion from the analysis and baseline comparisons between included and excluded participants

Table S1A Primary reasons for exclusion from the analysis and their distribution across follow-up stages

Primary reason	During cycle 1	Between cycles	During cycle 2	Final screening	Total
Withdrawal	14	0	10	0	24
Loss to follow-up	6	8	8	0	22
Change in the primary chemotherapy regimen	3	5	0	0	8
Discontinuation of chemotherapy	2	6	0	0	8
Transfer to another hospital	2	2	0	0	4
Death	0	1	0	0	1
Insufficient valid assessments	0	0	0	25	25
Total	27	22	18	25	92

Table S1B Baseline characteristics of participants included in and excluded from the analysis

Variable	Included (n = 110)	Excluded (n = 92)	SMD
Age (years)	61.60 ± 9.63	59.59 ± 9.64	0.209
BMI (kg/m²)	22.50 ± 3.17	22.22 ± 3.48	0.085
Albumin (g/L)	40.31 ± 4.14	39.68 ± 4.78	0.142
Hemoglobin (g/L)	120.81 ± 14.72	118.84 ± 13.75	0.138
NRS-2002 score	1.99 ± 0.89	2.04 ± 0.90	-0.059
Male	73(66.4%)	60(65.2%)	0.024
Stage III	76(69.1%)	61(66.3%)	0.060
At nutritional risk	25(22.7%)	27(29.3%)	-0.151
Stoma present	24(21.8%)	24(26.1%)	-0.100
Comorbidity present	43(39.1%)	37(40.2%)	-0.023
CAPOX	49(44.5%)	39(42.4%)	0.043
mFOLFOX6	42(38.2%)	37(40.2%)	-0.042
Capecitabine monotherapy	3(2.7%)	1(1.1%)	0.120
Other regimens	16(14.5%)	15(16.3%)	-0.049

Note: SMD denotes standardized mean difference and describes the magnitude of differences in baseline characteristics between included and excluded participants.

Table S2 EMA completion and missing assessments

Group	Scheduled assessments	Complete assessments	Missing assessments	Completion rate (%)
Overall	4620	4134	486	89.5
Cycle 1	2310	2090	220	90.5
Cycle 2	2310	2044	266	88.5
Morning	1540	1381	159	89.7
Afternoon	1540	1363	177	88.5
Evening	1540	1390	150	90.3
Day 1	660	590	70	89.4
Day 2	660	588	72	89.1
Day 3	660	595	65	90.2
Day 4	660	587	73	88.9
Day 5	660	603	57	91.4
Day 6	660	592	68	89.7
Day 7	660	579	81	87.7

Table S3 Mixed-effects logistic regression analyses of missing EMA assessments

Table S3A Factors associated with missingness across all scheduled EMA assessments

Variable	OR (95% CI)	P	Exploratory BH q
Cycle 2 vs cycle 1	1.241(1.027–1.499)	0.026	0.307
Afternoon vs morning	1.127(0.898–1.416)	0.302	0.725
Evening vs morning	0.932(0.735–1.181)	0.558	0.852
Scheduled time since chemotherapy (per 1-day increase)	1.010(0.963–1.059)	0.679	0.852
Age (per 10-year increase)	1.088(0.955–1.240)	0.204	0.725
Female vs male	1.023(0.788–1.328)	0.863	0.911
BMI (per 5 kg/m² increase)	0.952(0.780–1.162)	0.630	0.852
Comorbidity present vs absent	0.829(0.640–1.074)	0.156	0.725
Nutritional risk present vs absent	1.017(0.753–1.374)	0.911	0.911
mFOLFOX6 vs CAPOX	0.950(0.726–1.243)	0.710	0.852
Capecitabine monotherapy vs CAPOX	0.640(0.278–1.476)	0.295	0.725
Other regimens vs CAPOX	1.084(0.747–1.573)	0.672	0.852

Table S3B Factors associated with missingness in the subset with a complete preceding assessment

Variable	OR (95% CI)	P	Exploratory BH q
Cycle 2 vs cycle 1	1.292(1.048–1.594)	0.017	0.216
Afternoon vs morning	1.173(0.911–1.510)	0.217	0.846
Evening vs morning	0.952(0.733–1.238)	0.716	0.846
Scheduled time since chemotherapy (per 1-day increase)	1.007(0.953–1.064)	0.799	0.866
Age (per 10-year increase)	1.062(0.916–1.230)	0.426	0.846
Female vs male	1.110(0.829–1.486)	0.482	0.846
BMI (per 5 kg/m² increase)	0.955(0.763–1.195)	0.686	0.846
Comorbidity present vs absent	0.789(0.589–1.058)	0.113	0.735
Nutritional risk present vs absent	1.017(0.725–1.429)	0.921	0.921
mFOLFOX6 vs CAPOX	0.891(0.659–1.204)	0.451	0.846
Capecitabine monotherapy vs CAPOX	0.600(0.239–1.510)	0.278	0.846
Other regimens vs CAPOX	0.918(0.598–1.410)	0.696	0.846

Variable	OR (95% CI)	P	Exploratory BH q
Previous mean symptom burden (per 1 SD)	0.968(0.862–1.087)	0.583	0.846

Note: The outcome is a missing EMA assessment (1 = missing, 0 = complete). The models include a patient-level random intercept. BH q values represent exploratory adjustment for multiple testing.

Table S4 Estimated marginal means for 13 symptoms across time intervals in two chemotherapy cycles

Table S4A Estimated marginal means for 13 symptoms across time intervals in cycle 1

Symptom	0–24 h	>24–72 h	>72–120 h	>120–168 h
Nausea	1.93(1.80–2.06)	1.26(1.15–1.38)	0.94(0.83–1.05)	0.74(0.63–0.86)
Vomiting severity	0.30(0.25–0.36)	0.25(0.21–0.29)	0.28(0.24–0.32)	0.24(0.19–0.28)
Fatigue	3.19(2.98–3.40)	3.79(3.59–3.98)	3.76(3.57–3.95)	3.03(2.84–3.22)
Loss of appetite	1.95(1.78–2.11)	2.84(2.69–2.98)	2.48(2.34–2.62)	1.56(1.41–1.70)
Diarrhea	0.72(0.61–0.83)	1.07(0.97–1.16)	0.97(0.88–1.07)	0.63(0.53–0.72)
Constipation	0.65(0.54–0.76)	0.94(0.84–1.03)	1.20(1.11–1.29)	1.04(0.95–1.14)
Abdominal distension	1.03(0.93–1.14)	1.54(1.46–1.63)	1.55(1.46–1.63)	0.88(0.79–0.96)
Abdominal pain	1.01(0.89–1.14)	1.31(1.20–1.41)	1.25(1.15–1.35)	0.89(0.79–0.99)
Sleep disturbance	2.16(1.99–2.32)	2.70(2.55–2.85)	2.67(2.52–2.82)	2.00(1.85–2.15)
Anxiety	1.72(1.57–1.87)	1.50(1.37–1.64)	1.39(1.26–1.53)	1.33(1.19–1.47)
Depression	0.92(0.79–1.04)	0.97(0.86–1.09)	1.19(1.08–1.31)	1.33(1.22–1.45)
Dry mouth	1.30(1.20–1.41)	1.63(1.54–1.71)	1.22(1.14–1.31)	0.76(0.67–0.85)
Numbness/tingling	0.86(0.74–0.98)	0.77(0.67–0.88)	0.71(0.61–0.81)	0.87(0.77–0.98)

Table S4B Estimated marginal means for 13 symptoms across time intervals in cycle 2

Symptom	0–24 h	>24–72 h	>72–120 h	>120–168 h
Nausea	1.92(1.79–2.05)	1.25(1.14–1.36)	0.93(0.82–1.04)	0.73(0.62–0.85)
Vomiting severity	0.31(0.25–0.36)	0.26(0.21–0.30)	0.28(0.25–0.33)	0.24(0.19–0.28)
Fatigue	3.76(3.55–3.97)	4.35(4.16–4.54)	4.32(4.13–4.51)	3.60(3.41–3.79)
Loss of appetite	2.29(2.12–2.45)	3.18(3.03–3.32)	2.82(2.67–2.96)	1.89(1.75–2.04)
Diarrhea	0.76(0.65–0.86)	1.10(1.01–1.20)	1.01(0.91–1.10)	0.66(0.56–0.76)
Constipation	0.71(0.60–0.82)	1.00(0.90–1.09)	1.26(1.17–1.35)	1.10(1.01–1.20)
Abdominal distension	1.10(0.99–1.20)	1.60(1.52–1.69)	1.61(1.52–1.69)	0.94(0.85–1.03)
Abdominal pain	1.03(0.91–1.15)	1.33(1.22–1.43)	1.27(1.16–1.37)	0.91(0.80–1.01)
Sleep disturbance	2.55(2.38–2.72)	3.09(2.94–3.24)	3.06(2.91–3.21)	2.40(2.25–2.55)

Symptom	0–24 h	>24–72 h	>72–120 h	>120–168 h
Anxiety	1.64(1.48–1.79)	1.42(1.28–1.56)	1.31(1.18–1.44)	1.24(1.10–1.38)
Depression	0.95(0.82–1.08)	1.01(0.90–1.13)	1.23(1.12–1.34)	1.37(1.25–1.49)
Dry mouth	1.44(1.33–1.54)	1.76(1.67–1.85)	1.35(1.27–1.44)	0.89(0.81–0.98)
Numbness/tingling	1.36(1.24–1.48)	1.27(1.17–1.38)	1.21(1.11–1.31)	1.38(1.27–1.48)

Table S5 Estimated marginal symptom means and differences between two chemotherapy cycles

Symptom	Cycle 1 EMM (95% CI)	Cycle 2 EMM (95% CI)	Difference, cycle 2 − cycle 1 (95% CI)	P	FDR P
Nausea	1.12(1.02–1.22)	1.11(1.01–1.21)	-0.011(-0.077–0.056)	0.754	0.817
Vomiting severity	0.26(0.23–0.30)	0.27(0.23–0.30)	0.002(-0.031–0.034)	0.922	0.922
Fatigue	3.48(3.30–3.65)	4.04(3.87–4.22)	0.567(0.469–0.666)	<0.001	<0.001
Loss of appetite	2.24(2.11–2.37)	2.58(2.45–2.71)	0.338(0.248–0.428)	<0.001	<0.001
Diarrhea	0.87(0.78–0.95)	0.90(0.81–0.99)	0.033(-0.022–0.089)	0.239	0.311
Constipation	1.00(0.92–1.08)	1.06(0.98–1.14)	0.060(0.001–0.119)	0.046	0.085
Abdominal distension	1.28(1.21–1.36)	1.34(1.27–1.42)	0.062(-0.001–0.124)	0.053	0.085
Abdominal pain	1.13(1.04–1.22)	1.15(1.06–1.24)	0.019(-0.050–0.087)	0.592	0.699
Sleep disturbance	2.41(2.28–2.55)	2.81(2.67–2.94)	0.394(0.315–0.474)	<0.001	<0.001
Anxiety	1.45(1.33–1.58)	1.37(1.24–1.50)	-0.083(-0.150–-0.016)	0.015	0.033
Depression	1.13(1.02–1.23)	1.17(1.06–1.27)	0.038(-0.023–0.100)	0.222	0.311
Dry mouth	1.22(1.14–1.29)	1.35(1.27–1.43)	0.132(0.072–0.191)	<0.001	<0.001
Numbness/tingling	0.79(0.70–0.89)	1.30(1.21–1.39)	0.504(0.441–0.566)	<0.001	<0.001

Note: EMM denotes estimated marginal mean. P values are adjusted across the 13 symptoms using the Benjamini–Hochberg procedure.

Table S6 All 156 cross-symptom predictive coefficients in the primary temporal network

Symptom at t−1	Symptom at t	β	SE	95% Wald CI	95% bootstrap CI	P	BH q
Nausea	Vomiting severity	0.077	0.022	0.035–0.119	0.028–0.113	<0.001	0.003
Nausea	Fatigue	0.036	0.015	0.006–0.066	0.003–0.060	0.018	0.086
Nausea	Loss of appetite	0.100	0.016	0.069–0.132	0.075–0.124	<0.001	<0.001
Nausea	Diarrhea	0.007	0.017	-0.026–0.040	-0.029–0.039	0.685	0.862
Nausea	Constipation	-0.055	0.017	-0.089–-0.021	-0.088–-0.023	0.001	0.011
Nausea	Abdominal distension	0.010	0.018	-0.024–0.045	-0.018–0.046	0.552	0.812
Nausea	Abdominal pain	0.024	0.018	-0.012–0.059	-0.007–0.052	0.193	0.451

Symptom at t−1	Symptom at t	β	SE	95% Wald CI	95% bootstrap CI	P	BH q
Nausea	Sleep disturbance	-0.017	0.016	-0.049–0.014	-0.048–0.018	0.280	0.574
Nausea	Anxiety	-0.003	0.016	-0.034–0.029	-0.034–0.026	0.861	0.960
Nausea	Depression	-0.055	0.017	-0.087–-0.022	-0.083–-0.031	<0.001	0.007
Nausea	Dry mouth	0.055	0.017	0.020–0.089	0.025–0.087	0.002	0.012
Nausea	Numbness/tingling	-0.018	0.018	-0.053–0.018	-0.052–0.021	0.322	0.627
Vomiting severity	Nausea	-0.002	0.017	-0.035–0.031	-0.034–0.030	0.915	0.965
Vomiting severity	Fatigue	-0.013	0.014	-0.040–0.014	-0.038–0.015	0.341	0.649
Vomiting severity	Loss of appetite	-0.000	0.015	-0.030–0.029	-0.035–0.026	0.979	0.989
Vomiting severity	Diarrhea	0.011	0.017	-0.022–0.044	-0.020–0.050	0.520	0.803
Vomiting severity	Constipation	-0.032	0.017	-0.065–0.001	-0.067–0.003	0.058	0.214
Vomiting severity	Abdominal distension	-0.021	0.018	-0.055–0.014	-0.055–0.014	0.237	0.506
Vomiting severity	Abdominal pain	-0.028	0.017	-0.060–0.005	-0.057–0.008	0.096	0.301
Vomiting severity	Sleep disturbance	-0.022	0.015	-0.051–0.008	-0.057–0.004	0.149	0.404
Vomiting severity	Anxiety	-0.001	0.017	-0.033–0.032	-0.032–0.028	0.967	0.989
Vomiting severity	Depression	-0.011	0.015	-0.042–0.019	-0.036–0.017	0.463	0.761
Vomiting severity	Dry mouth	-0.032	0.018	-0.066–0.003	-0.067–0.003	0.075	0.239
Vomiting severity	Numbness/tingling	-0.006	0.019	-0.043–0.031	-0.042–0.028	0.740	0.894
Fatigue	Nausea	-0.005	0.021	-0.046–0.036	-0.047–0.030	0.827	0.928
Fatigue	Vomiting severity	0.027	0.021	-0.014–0.069	-0.013–0.069	0.200	0.453
Fatigue	Loss of appetite	0.052	0.019	0.015–0.089	0.018–0.086	0.006	0.031
Fatigue	Diarrhea	0.013	0.019	-0.025–0.051	-0.023–0.049	0.493	0.782
Fatigue	Constipation	0.023	0.022	-0.019–0.066	-0.017–0.064	0.283	0.574
Fatigue	Abdominal distension	0.033	0.020	-0.007–0.072	-0.005–0.075	0.106	0.323
Fatigue	Abdominal pain	0.010	0.019	-0.028–0.048	-0.023–0.049	0.603	0.848
Fatigue	Sleep disturbance	0.074	0.020	0.036–0.113	0.039–0.110	<0.001	0.002
Fatigue	Anxiety	-0.002	0.019	-0.038–0.034	-0.034–0.032	0.908	0.965
Fatigue	Depression	0.034	0.019	-0.003–0.071	-0.007–0.075	0.074	0.239
Fatigue	Dry mouth	0.046	0.020	0.007–0.085	0.009–0.087	0.021	0.096
Fatigue	Numbness/tingling	-0.013	0.019	-0.051–0.025	-0.047–0.022	0.493	0.782
Loss of appetite	Nausea	0.014	0.018	-0.020–0.049	-0.021–0.042	0.417	0.732

Symptom at t−1	Symptom at t	β	SE	95% Wald CI	95% bootstrap CI	P	BH q
Loss of appetite	Vomiting severity	0.015	0.019	-0.022–0.052	-0.018–0.048	0.438	0.746
Loss of appetite	Fatigue	0.123	0.015	0.093–0.153	0.097–0.147	<0.001	<0.001
Loss of appetite	Diarrhea	0.069	0.018	0.034–0.104	0.040–0.098	<0.001	0.001
Loss of appetite	Constipation	0.021	0.019	-0.016–0.058	-0.009–0.055	0.258	0.545
Loss of appetite	Abdominal distension	0.110	0.020	0.070–0.150	0.068–0.144	<0.001	<0.001
Loss of appetite	Abdominal pain	0.078	0.018	0.043–0.113	0.048–0.110	<0.001	<0.001
Loss of appetite	Sleep disturbance	0.105	0.017	0.072–0.139	0.078–0.137	<0.001	<0.001
Loss of appetite	Anxiety	-0.011	0.017	-0.044–0.023	-0.040–0.026	0.535	0.804
Loss of appetite	Depression	-0.000	0.019	-0.037–0.036	-0.041–0.035	0.983	0.989
Loss of appetite	Dry mouth	0.077	0.019	0.040–0.113	0.041–0.109	<0.001	<0.001
Loss of appetite	Numbness/tingling	-0.006	0.018	-0.041–0.029	-0.036–0.031	0.745	0.894
Diarrhea	Nausea	0.002	0.017	-0.031–0.034	-0.027–0.030	0.927	0.966
Diarrhea	Vomiting severity	0.014	0.018	-0.021–0.049	-0.018–0.049	0.448	0.752
Diarrhea	Fatigue	0.026	0.015	-0.002–0.055	-0.000–0.050	0.072	0.239
Diarrhea	Loss of appetite	0.046	0.016	0.014–0.078	0.018–0.077	0.005	0.027
Diarrhea	Constipation	0.015	0.018	-0.021–0.051	-0.015–0.046	0.418	0.732
Diarrhea	Abdominal distension	0.016	0.018	-0.018–0.051	-0.013–0.045	0.354	0.657
Diarrhea	Abdominal pain	0.007	0.017	-0.027–0.041	-0.027–0.041	0.690	0.862
Diarrhea	Sleep disturbance	0.018	0.016	-0.014–0.050	-0.008–0.048	0.271	0.564
Diarrhea	Anxiety	0.002	0.016	-0.030–0.034	-0.025–0.035	0.888	0.965
Diarrhea	Depression	-0.036	0.017	-0.069–-0.003	-0.064–-0.003	0.032	0.135
Diarrhea	Dry mouth	0.038	0.018	0.003–0.074	0.005–0.073	0.035	0.141
Diarrhea	Numbness/tingling	-0.023	0.017	-0.056–0.011	-0.050–0.008	0.182	0.438
Constipation	Nausea	-0.022	0.016	-0.054–0.010	-0.053–0.013	0.178	0.435
Constipation	Vomiting severity	0.005	0.019	-0.032–0.041	-0.029–0.037	0.798	0.902
Constipation	Fatigue	0.002	0.015	-0.026–0.031	-0.028–0.028	0.880	0.965
Constipation	Loss of appetite	-0.019	0.016	-0.050–0.012	-0.047–0.012	0.237	0.506
Constipation	Diarrhea	-0.017	0.017	-0.049–0.016	-0.047–0.015	0.325	0.627
Constipation	Abdominal distension	-0.001	0.017	-0.035–0.034	-0.029–0.034	0.976	0.989
Constipation	Abdominal pain	0.010	0.017	-0.023–0.043	-0.019–0.037	0.541	0.804

Symptom at t−1	Symptom at t	β	SE	95% Wald CI	95% bootstrap CI	P	BH q
Constipation	Sleep disturbance	-0.019	0.016	-0.051–0.012	-0.051–0.012	0.233	0.506
Constipation	Anxiety	0.010	0.016	-0.022–0.041	-0.020–0.038	0.540	0.804
Constipation	Depression	0.036	0.017	0.003–0.070	0.002–0.067	0.035	0.141
Constipation	Dry mouth	-0.054	0.017	-0.088–-0.020	-0.080–-0.026	0.002	0.011
Constipation	Numbness/tingling	0.017	0.017	-0.016–0.050	-0.015–0.047	0.306	0.605
Abdominal distension	Nausea	0.008	0.016	-0.024–0.039	-0.022–0.044	0.645	0.862
Abdominal distension	Vomiting severity	-0.005	0.017	-0.040–0.029	-0.035–0.028	0.759	0.894
Abdominal distension	Fatigue	0.026	0.014	-0.002–0.054	-0.003–0.053	0.070	0.239
Abdominal distension	Loss of appetite	0.087	0.017	0.054–0.120	0.051–0.120	<0.001	<0.001
Abdominal distension	Diarrhea	0.050	0.018	0.016–0.085	0.011–0.081	0.004	0.027
Abdominal distension	Constipation	0.033	0.018	-0.003–0.069	0.001–0.067	0.071	0.239
Abdominal distension	Abdominal pain	0.062	0.017	0.029–0.094	0.032–0.092	<0.001	0.002
Abdominal distension	Sleep disturbance	0.011	0.016	-0.020–0.042	-0.022–0.043	0.496	0.782
Abdominal distension	Anxiety	0.011	0.016	-0.020–0.042	-0.020–0.040	0.477	0.775
Abdominal distension	Depression	0.002	0.016	-0.030–0.034	-0.029–0.029	0.896	0.965
Abdominal distension	Dry mouth	0.034	0.018	-0.001–0.069	0.002–0.072	0.054	0.207
Abdominal distension	Numbness/tingling	-0.011	0.018	-0.047–0.024	-0.054–0.022	0.530	0.804
Abdominal pain	Nausea	-0.014	0.017	-0.046–0.019	-0.044–0.020	0.415	0.732
Abdominal pain	Vomiting severity	-0.006	0.018	-0.041–0.029	-0.040–0.031	0.749	0.894
Abdominal pain	Fatigue	0.022	0.015	-0.007–0.051	-0.004–0.046	0.136	0.387
Abdominal pain	Loss of appetite	0.036	0.016	0.005–0.068	0.005–0.073	0.025	0.112
Abdominal pain	Diarrhea	0.035	0.017	0.001–0.069	-0.002–0.065	0.043	0.168
Abdominal pain	Constipation	-0.007	0.018	-0.042–0.027	-0.032–0.022	0.684	0.862
Abdominal pain	Abdominal distension	0.057	0.018	0.022–0.092	0.027–0.090	0.001	0.010
Abdominal pain	Sleep disturbance	0.043	0.018	0.008–0.078	0.015–0.072	0.016	0.079
Abdominal pain	Anxiety	-0.014	0.016	-0.046–0.018	-0.047–0.016	0.385	0.707
Abdominal pain	Depression	-0.013	0.017	-0.046–0.020	-0.041–0.015	0.432	0.746
Abdominal pain	Dry mouth	0.002	0.017	-0.032–0.036	-0.030–0.034	0.911	0.965
Abdominal pain	Numbness/tingling	-0.002	0.019	-0.039–0.036	-0.032–0.039	0.935	0.966
Sleep disturbance	Nausea	-0.024	0.018	-0.059–0.010	-0.056–0.005	0.167	0.435

Symptom at t−1	Symptom at t	β	SE	95% Wald CI	95% bootstrap CI	P	BH q
Sleep disturbance	Vomiting severity	-0.026	0.019	-0.063–0.011	-0.056–0.007	0.175	0.435
Sleep disturbance	Fatigue	0.102	0.016	0.071–0.133	0.073–0.130	<0.001	<0.001
Sleep disturbance	Loss of appetite	0.032	0.017	-0.002–0.066	-0.002–0.063	0.062	0.224
Sleep disturbance	Diarrhea	0.009	0.018	-0.026–0.044	-0.027–0.045	0.613	0.848
Sleep disturbance	Constipation	0.013	0.020	-0.026–0.053	-0.024–0.052	0.501	0.782
Sleep disturbance	Abdominal distension	0.054	0.019	0.016–0.093	0.014–0.086	0.005	0.029
Sleep disturbance	Abdominal pain	-0.002	0.020	-0.041–0.037	-0.040–0.047	0.933	0.966
Sleep disturbance	Anxiety	-0.009	0.017	-0.042–0.025	-0.039–0.027	0.620	0.848
Sleep disturbance	Depression	0.057	0.018	0.023–0.092	0.026–0.092	0.001	0.009
Sleep disturbance	Dry mouth	0.028	0.019	-0.008–0.065	-0.009–0.066	0.127	0.372
Sleep disturbance	Numbness/tingling	0.007	0.018	-0.028–0.042	-0.025–0.047	0.681	0.862
Anxiety	Nausea	0.042	0.019	0.005–0.079	0.007–0.077	0.027	0.119
Anxiety	Vomiting severity	0.032	0.022	-0.011–0.075	-0.009–0.068	0.150	0.404
Anxiety	Fatigue	-0.004	0.015	-0.034–0.026	-0.033–0.019	0.789	0.899
Anxiety	Loss of appetite	-0.007	0.017	-0.040–0.026	-0.041–0.023	0.676	0.862
Anxiety	Diarrhea	-0.015	0.020	-0.054–0.024	-0.059–0.024	0.440	0.746
Anxiety	Constipation	0.002	0.018	-0.034–0.038	-0.032–0.037	0.904	0.965
Anxiety	Abdominal distension	0.006	0.019	-0.031–0.042	-0.028–0.040	0.763	0.894
Anxiety	Abdominal pain	-0.009	0.018	-0.044–0.026	-0.040–0.020	0.614	0.848
Anxiety	Sleep disturbance	0.077	0.021	0.036–0.117	0.043–0.115	<0.001	0.002
Anxiety	Depression	0.008	0.018	-0.028–0.044	-0.034–0.043	0.673	0.862
Anxiety	Dry mouth	0.024	0.018	-0.012–0.060	-0.011–0.057	0.194	0.451
Anxiety	Numbness/tingling	0.006	0.020	-0.034–0.045	-0.030–0.046	0.768	0.894
Depression	Nausea	-0.033	0.018	-0.069–0.003	-0.064–0.001	0.069	0.239
Depression	Vomiting severity	-0.006	0.018	-0.042–0.030	-0.040–0.026	0.742	0.894
Depression	Fatigue	-0.019	0.015	-0.048–0.011	-0.045–0.007	0.222	0.495
Depression	Loss of appetite	-0.023	0.017	-0.056–0.010	-0.048–0.004	0.174	0.435
Depression	Diarrhea	-0.005	0.018	-0.039–0.029	-0.036–0.030	0.776	0.896
Depression	Constipation	0.008	0.018	-0.028–0.043	-0.029–0.035	0.663	0.862
Depression	Abdominal distension	-0.027	0.018	-0.063–0.009	-0.055–0.002	0.139	0.387

Symptom at t−1	Symptom at t	β	SE	95% Wald CI	95% bootstrap CI	P	BH q
Depression	Abdominal pain	-0.025	0.018	-0.059–0.010	-0.060–0.006	0.162	0.428
Depression	Sleep disturbance	-0.027	0.017	-0.061–0.006	-0.061–0.004	0.113	0.339
Depression	Anxiety	0.000	0.017	-0.033–0.033	-0.030–0.029	0.991	0.991
Depression	Dry mouth	-0.049	0.020	-0.089–-0.010	-0.097–-0.013	0.013	0.067
Depression	Numbness/tingling	0.014	0.017	-0.020–0.049	-0.014–0.047	0.410	0.732
Dry mouth	Nausea	0.021	0.016	-0.011–0.053	-0.009–0.048	0.198	0.453
Dry mouth	Vomiting severity	-0.010	0.019	-0.047–0.027	-0.044–0.024	0.600	0.848
Dry mouth	Fatigue	0.058	0.014	0.029–0.086	0.030–0.087	<0.001	<0.001
Dry mouth	Loss of appetite	0.083	0.016	0.051–0.114	0.055–0.112	<0.001	<0.001
Dry mouth	Diarrhea	0.085	0.018	0.050–0.119	0.054–0.111	<0.001	<0.001
Dry mouth	Constipation	-0.002	0.017	-0.036–0.031	-0.033–0.025	0.891	0.965
Dry mouth	Abdominal distension	0.082	0.017	0.048–0.116	0.052–0.115	<0.001	<0.001
Dry mouth	Abdominal pain	0.072	0.017	0.038–0.105	0.041–0.103	<0.001	<0.001
Dry mouth	Sleep disturbance	0.008	0.016	-0.023–0.039	-0.025–0.037	0.605	0.848
Dry mouth	Anxiety	0.016	0.017	-0.017–0.050	-0.015–0.051	0.345	0.649
Dry mouth	Depression	-0.046	0.017	-0.079–-0.013	-0.074–-0.013	0.007	0.034
Dry mouth	Numbness/tingling	-0.010	0.018	-0.045–0.026	-0.051–0.024	0.602	0.848
Numbness/tingling	Nausea	-0.025	0.018	-0.061–0.011	-0.057–0.012	0.176	0.435
Numbness/tingling	Vomiting severity	-0.010	0.019	-0.048–0.027	-0.050–0.023	0.591	0.848
Numbness/tingling	Fatigue	0.043	0.015	0.014–0.072	0.015–0.070	0.004	0.022
Numbness/tingling	Loss of appetite	0.025	0.016	-0.007–0.057	-0.004–0.054	0.129	0.372
Numbness/tingling	Diarrhea	0.005	0.017	-0.029–0.038	-0.034–0.038	0.781	0.896
Numbness/tingling	Constipation	0.007	0.018	-0.027–0.042	-0.025–0.039	0.685	0.862
Numbness/tingling	Abdominal distension	0.006	0.019	-0.032–0.044	-0.028–0.045	0.757	0.894
Numbness/tingling	Abdominal pain	0.008	0.017	-0.026–0.041	-0.024–0.040	0.658	0.862
Numbness/tingling	Sleep disturbance	0.017	0.016	-0.015–0.049	-0.015–0.046	0.301	0.602
Numbness/tingling	Anxiety	-0.012	0.017	-0.045–0.020	-0.043–0.016	0.456	0.757
Numbness/tingling	Depression	0.006	0.019	-0.032–0.044	-0.029–0.039	0.751	0.894
Numbness/tingling	Dry mouth	-0.007	0.018	-0.041–0.027	-0.042–0.021	0.690	0.862

Table S7 All 78 pairwise conditional associations in the primary contemporaneous network

Symptom 1	Symptom 2	Partial r	95% bootstrap CI
Fatigue	Sleep disturbance	0.144	0.107–0.176
Abdominal distension	Abdominal pain	0.144	0.110–0.170
Anxiety	Depression	0.135	0.093–0.174
Loss of appetite	Dry mouth	0.107	0.082–0.134
Fatigue	Loss of appetite	0.103	0.063–0.133
Nausea	Vomiting severity	0.093	0.064–0.127
Nausea	Loss of appetite	0.092	0.061–0.126
Nausea	Dry mouth	0.082	0.046–0.117
Loss of appetite	Sleep disturbance	0.081	0.047–0.110
Diarrhea	Abdominal pain	0.068	0.027–0.103
Constipation	Abdominal distension	0.067	0.036–0.099
Diarrhea	Abdominal distension	0.064	0.033–0.095
Constipation	Abdominal pain	0.063	0.028–0.093
Sleep disturbance	Depression	0.061	0.020–0.099
Loss of appetite	Abdominal distension	0.057	0.027–0.090
Sleep disturbance	Anxiety	0.057	0.024–0.089
Diarrhea	Depression	-0.045	-0.072–-0.009
Constipation	Numbness/tingling	-0.044	-0.075–-0.010
Fatigue	Constipation	0.037	0.006–0.080
Fatigue	Abdominal distension	0.037	0.007–0.068
Nausea	Numbness/tingling	-0.037	-0.063–-0.007
Diarrhea	Dry mouth	0.036	-0.003–0.075
Vomiting severity	Constipation	0.032	0.002–0.063
Nausea	Constipation	-0.032	-0.062–-0.000
Vomiting severity	Loss of appetite	0.028	0.003–0.055
Loss of appetite	Depression	-0.028	-0.061–0.003
Loss of appetite	Diarrhea	0.027	-0.010–0.056
Vomiting severity	Dry mouth	0.027	-0.006–0.057
Sleep disturbance	Dry mouth	0.027	-0.011–0.058
Diarrhea	Numbness/tingling	-0.026	-0.058–0.005
Nausea	Abdominal pain	0.026	-0.005–0.061
Loss of appetite	Numbness/tingling	0.026	-0.012–0.059
Nausea	Anxiety	0.025	-0.005–0.059
Nausea	Fatigue	-0.024	-0.057–0.008
Fatigue	Diarrhea	0.023	-0.012–0.055
Abdominal distension	Sleep disturbance	0.022	-0.008–0.050
Vomiting severity	Diarrhea	-0.022	-0.055–0.005
Abdominal distension	Dry mouth	0.022	-0.015–0.053
Anxiety	Numbness/tingling	-0.020	-0.049–0.011
Abdominal distension	Numbness/tingling	-0.020	-0.053–0.015
Constipation	Anxiety	-0.019	-0.050–0.012

Symptom 1	Symptom 2	Partial r	95% bootstrap CI
Nausea	Sleep disturbance	0.017	-0.013–0.048
Sleep disturbance	Numbness/tingling	0.017	-0.017–0.051
Abdominal distension	Depression	-0.017	-0.047–0.015
Constipation	Depression	0.017	-0.012–0.045
Anxiety	Dry mouth	0.016	-0.008–0.050
Loss of appetite	Anxiety	0.016	-0.014–0.048
Nausea	Depression	-0.016	-0.047–0.015
Loss of appetite	Constipation	-0.016	-0.049–0.017
Diarrhea	Constipation	0.016	-0.013–0.046
Fatigue	Numbness/tingling	0.015	-0.022–0.047
Diarrhea	Sleep disturbance	0.014	-0.011–0.053
Vomiting severity	Numbness/tingling	0.013	-0.025–0.053
Vomiting severity	Anxiety	-0.012	-0.041–0.013
Depression	Dry mouth	-0.012	-0.045–0.017
Vomiting severity	Depression	-0.011	-0.034–0.013
Abdominal pain	Depression	-0.010	-0.038–0.017
Abdominal pain	Anxiety	0.010	-0.030–0.046
Nausea	Diarrhea	-0.009	-0.042–0.023
Depression	Numbness/tingling	-0.008	-0.037–0.019
Fatigue	Abdominal pain	0.008	-0.028–0.043
Dry mouth	Numbness/tingling	0.006	-0.028–0.044
Fatigue	Depression	-0.006	-0.040–0.032
Diarrhea	Anxiety	0.005	-0.034–0.033
Vomiting severity	Abdominal distension	-0.005	-0.040–0.028
Vomiting severity	Sleep disturbance	-0.005	-0.035–0.024
Abdominal pain	Dry mouth	-0.005	-0.038–0.032
Loss of appetite	Abdominal pain	-0.004	-0.039–0.030
Nausea	Abdominal distension	0.004	-0.028–0.040
Fatigue	Dry mouth	0.004	-0.031–0.042
Constipation	Sleep disturbance	0.003	-0.026–0.033
Abdominal pain	Numbness/tingling	0.002	-0.030–0.031
Abdominal distension	Anxiety	0.002	-0.030–0.034
Fatigue	Anxiety	0.001	-0.034–0.037
Constipation	Dry mouth	-0.001	-0.033–0.033
Abdominal pain	Sleep disturbance	-0.001	-0.039–0.030
Vomiting severity	Abdominal pain	0.001	-0.026–0.031
Vomiting severity	Fatigue	-0.000	-0.030–0.030

Table S8 Bootstrap centrality and case-dropping stability analyses

Table S8A Correlations between centrality estimates after case dropping and those from the complete sample

Deletion proportion (%)	Patients retained	Measure	Median Pearson r	5th percentile of Pearson r	Median Spearman ρ	5th percentile of Spearman ρ
10.0	99	Temporal out-strength	0.990	0.982	0.967	0.918
10.0	99	Temporal in-strength	0.993	0.985	0.984	0.962
10.0	99	Contemporaneous strength	0.983	0.967	0.940	0.868
50.0	55	Temporal out-strength	0.930	0.857	0.857	0.735
50.0	55	Temporal in-strength	0.933	0.869	0.929	0.846
50.0	55	Contemporaneous strength	0.874	0.757	0.808	0.631

Table S8B Bootstrap distributions of centrality rankings

Symptom	Temporal out-strength rank (95% range)	Temporal out-strength ranked 1st (%)	Temporal in-strength rank (95% range)	Temporal in-strength ranked 1st (%)	Contemporaneous strength rank (95% range)	Contemporaneous strength ranked 1st (%)
Nausea	2.0–6.0	1.0	7.0–13.0	0.0	1.0–9.0	3.5
Vomiting severity	7.0–13.0	0.0	7.0–13.0	0.0	7.0–13.0	0.0
Fatigue	3.0–10.0	0.0	1.0–6.0	13.5	2.0–10.0	1.0
Loss of appetite	1.0–2.0	95.5	1.0–4.0	59.0	1.0–2.0	88.0
Diarrhea	6.0–13.0	0.0	4.0–10.0	0.0	5.0–12.0	0.0
Constipation	6.0–13.0	0.0	7.0–13.0	0.0	5.0–13.0	0.0
Abdominal distension	3.0–10.0	0.0	1.0–6.0	8.0	2.0–7.0	2.5
Abdominal pain	5.0–13.0	0.0	4.0–9.0	0.0	3.0–11.0	0.0
Sleep disturbance	2.0–8.0	0.0	1.0–8.0	10.0	1.0–8.0	5.0
Anxiety	6.0–13.0	0.0	11.0–13.0	0.0	4.0–13.0	0.0
Depression	5.0–13.0	0.0	5.0–10.0	0.0	4.0–12.0	0.0
Dry mouth	1.0–4.0	3.5	1.0–7.0	9.5	3.0–12.0	0.0
Numbness/tingling	7.0–13.0	0.0	9.0–13.0	0.0	9.0–13.0	0.0

Table S9 Overall dynamic network metrics for two chemotherapy cycles

Network	Cycle 1 global strength	Cycle 2 global strength	Difference, cycle 2 − cycle 1	Edge-weight correlation r
Temporal	4.699	4.808	0.109	0.663
Contemporaneous	2.737	2.533	-0.204	0.697

Table S10 Temporal and contemporaneous associations with the largest local edge-weight changes between two chemotherapy cycles

Table S10A The 10 temporal edges with the largest changes between cycles

Symptom at t−1	Symptom at t	Cycle 1 β	Cycle 2 β	Difference, cycle 2 − cycle 1
Constipation	Dry mouth	-0.012	-0.107	-0.095
Fatigue	Dry mouth	-0.003	0.087	0.090
Fatigue	Nausea	-0.053	0.032	0.085
Vomiting severity	Anxiety	0.036	-0.047	-0.083
Depression	Fatigue	0.018	-0.062	-0.080
Nausea	Abdominal distension	0.048	-0.031	-0.079
Fatigue	Anxiety	-0.037	0.035	0.072
Abdominal pain	Diarrhea	0.066	0.001	-0.065
Dry mouth	Diarrhea	0.055	0.119	0.064
Anxiety	Numbness/tingling	0.046	-0.017	-0.063

Table S10B The 10 contemporaneous edges with the largest changes between cycles

Symptom 1	Symptom 2	Cycle 1 r	Cycle 2 r	Difference, cycle 2 − cycle 1
Constipation	Abdominal distension	0.090	0.006	-0.084
Nausea	Loss of appetite	0.111	0.034	-0.077
Loss of appetite	Diarrhea	0.064	-0.010	-0.074
Constipation	Sleep disturbance	-0.032	0.041	0.073
Constipation	Dry mouth	0.030	-0.042	-0.071
Anxiety	Depression	0.160	0.091	-0.070
Fatigue	Abdominal pain	0.036	-0.031	-0.068
Vomiting severity	Numbness/tingling	-0.019	0.039	0.057
Loss of appetite	Abdominal distension	0.026	0.080	0.054

Symptom 1	Symptom 2	Cycle 1 r	Cycle 2 r	Difference, cycle 2 − cycle 1
Abdominal distension	Dry mouth	-0.004	0.049	0.053

Table S11 Clinical covariate adjustment and sensitivity analyses

Table S11A Estimates of key temporal associations across models

Temporal association	Primary model β	Clinically adjusted β (95% CI)	91-patient analysis β (95% CI)	Within-day 6 h analysis β (95% CI)
Loss of appetite → Fatigue	0.123	0.077(0.044–0.111)	0.128(0.094–0.161)	0.102(0.066–0.137)
Loss of appetite → Abdominal distension	0.110	0.027(-0.012–0.065)	0.115(0.071–0.158)	0.106(0.062–0.150)
Loss of appetite → Sleep disturbance	0.105	0.042(0.008–0.076)	0.104(0.067–0.141)	0.102(0.062–0.142)
Sleep disturbance → Fatigue	0.102	0.077(0.044–0.111)	0.111(0.078–0.145)	0.098(0.061–0.135)
Nausea → Loss of appetite	0.100	0.086(0.053–0.120)	0.102(0.068–0.137)	0.117(0.079–0.155)
Dry mouth → Diarrhea	0.085	0.048(0.013–0.083)	0.087(0.049–0.125)	0.080(0.037–0.124)
Dry mouth → Loss of appetite	0.083	0.009(-0.025–0.042)	0.084(0.050–0.118)	0.066(0.029–0.104)
Dry mouth → Abdominal distension	0.082	0.010(-0.024–0.044)	0.080(0.043–0.116)	0.072(0.031–0.113)

Table S11B Overall comparisons of sensitivity-analysis networks with the primary network

Model	Patients	Lagged Pairs	Temporal r	Contemporaneous r	Temporal Strength	Contemporaneous Strength
Time- and Supportive Medication-Adjusted Residuals	110	3354	0.6945	0.8957	2.590	1.912
Detrended Residuals	110	3354	0.7129	0.8947	2.598	1.951
±60-min Window Only	110	3096	0.9833	0.9903	4.258	2.465
Temporal Random Slopes Removed	110	3528	0.9991	0.9991	4.113	2.466
Vomiting Severity Node Removed	110	3528	0.9997	0.9996	3.716	2.205
Extended Clinical Covariate-Adjusted Residuals	110	3354	0.7002	0.8955	2.588	1.959
Restricted to CAPOX & mFOLFOX6	91	2916	0.9765	0.9822	4.504	2.526
Planned Within-Day 6-h Intervals Only	110	2452	0.9367	0.9527	4.450	2.605

Table S12 VAR(1) versus VAR(2) comparisons and a summary of mlVAR model size

Table S12A Comparison of VAR(1) and VAR(2) model fit using the same observations

Symptom	AIC1	AIC2	ΔAIC	BIC1	BIC2	ΔBIC	LRT χ^2	P	BH q
Nausea	7840.2	7845.2	4.99	8008.6	8091.7	83.16	21.01	0.073	0.105
Vomiting severity	8256.7	8267.6	10.89	8425.1	8514.1	89.07	15.11	0.301	0.326

Symptom	AIC1	AIC2	ΔAIC	BIC1	BIC2	ΔBIC	LRT χ^2	P	BH q
Fatigue	7291.8	7251.4	-40.41	7460.2	7498.0	37.76	66.41	<0.001	<0.001
Loss of appetite	7753.3	7714.7	-38.63	7921.7	7961.2	39.54	64.63	<0.001	<0.001
Diarrhea	8217.5	8216.0	-1.47	8385.9	8462.6	76.71	27.47	0.011	0.020
Constipation	8294.3	8277.9	-16.46	8462.7	8524.4	61.72	42.46	<0.001	<0.001
Abdominal distension	8303.4	8284.4	-18.93	8471.7	8531.0	59.24	44.93	<0.001	<0.001
Abdominal pain	8180.2	8190.4	10.24	8348.5	8437.0	88.42	15.76	0.263	0.310
Sleep disturbance	7885.2	7865.9	-19.29	8053.6	8112.5	58.88	45.29	<0.001	<0.001
Anxiety	7794.2	7802.1	7.97	7962.6	8048.7	86.14	18.03	0.156	0.203
Depression	7997.0	8010.6	13.60	8165.4	8257.2	91.77	12.40	0.495	0.495
Dry mouth	8296.1	8275.0	-21.10	8464.5	8521.6	57.08	47.10	<0.001	<0.001
Numbness/tingling	8200.0	8202.6	2.63	8368.3	8449.1	80.80	23.37	0.037	0.061

Table S12B Summary of mlVAR model size and fit

Model	Patients	Valid lagged pairs	Node equations	Fixed coefficients per equation	Random-effects parameters	Singular equations	Optimizer code = 0	Observations per fixed coefficient
Primary mlVAR model	110	3528	13	26	14	13/13	13/13	135.7
Residuals with expanded clinical covariate adjustment	110	3354	13	26	—	13/13	13/13	129.0
Restricted to CAPOX and mFOLFOX6	91	2916	13	26	—	13/13	13/13	112.2
Restricted to scheduled within-day 6 h intervals	110	2452	13	26	—	13/13	13/13	94.3

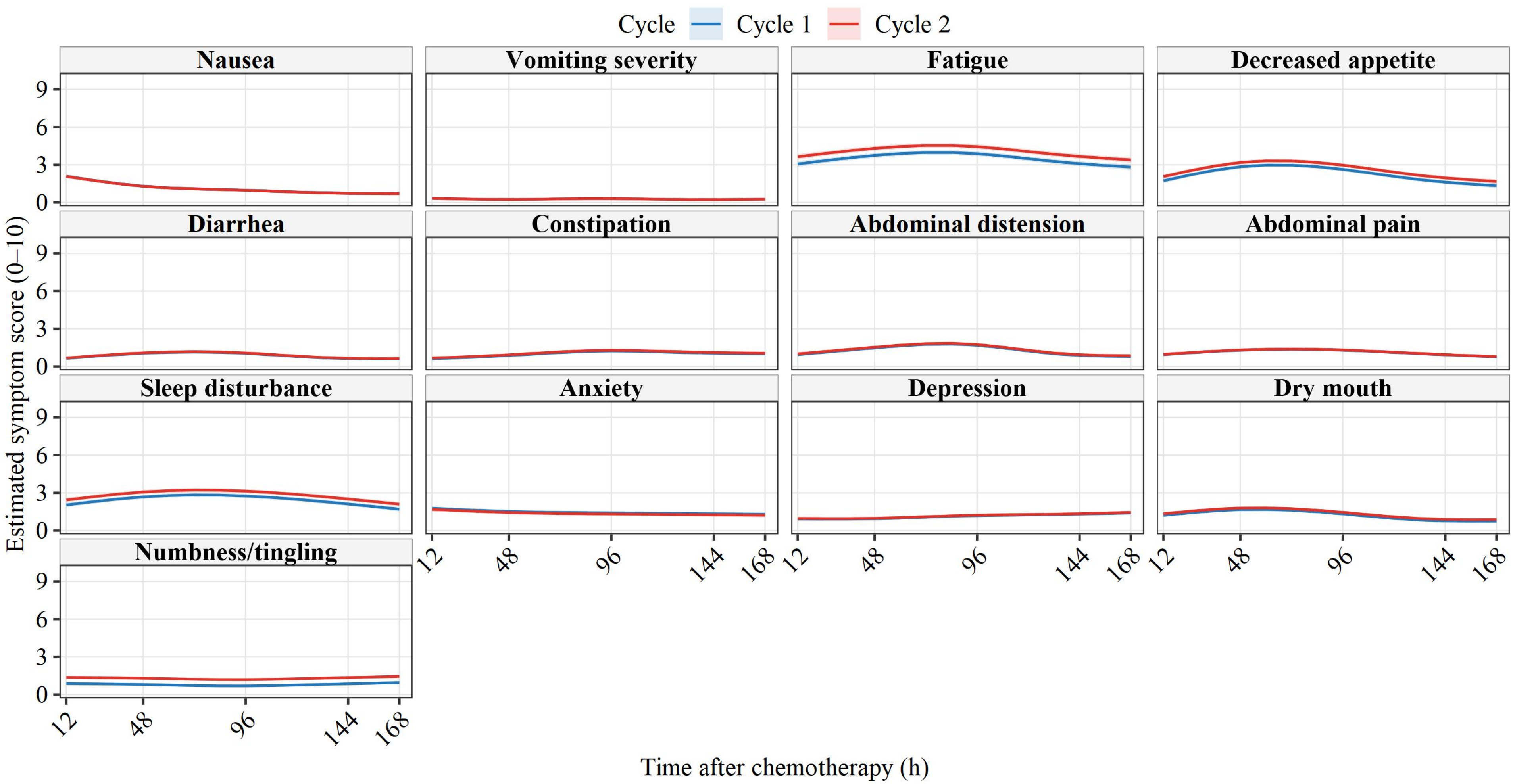

Figure S1 Trajectories of 13 symptoms on a common scale

Blue represents cycle 1 and red represents cycle 2; shaded areas indicate model-based 95% CIs. A common vertical scale facilitates comparison of absolute symptom burden.

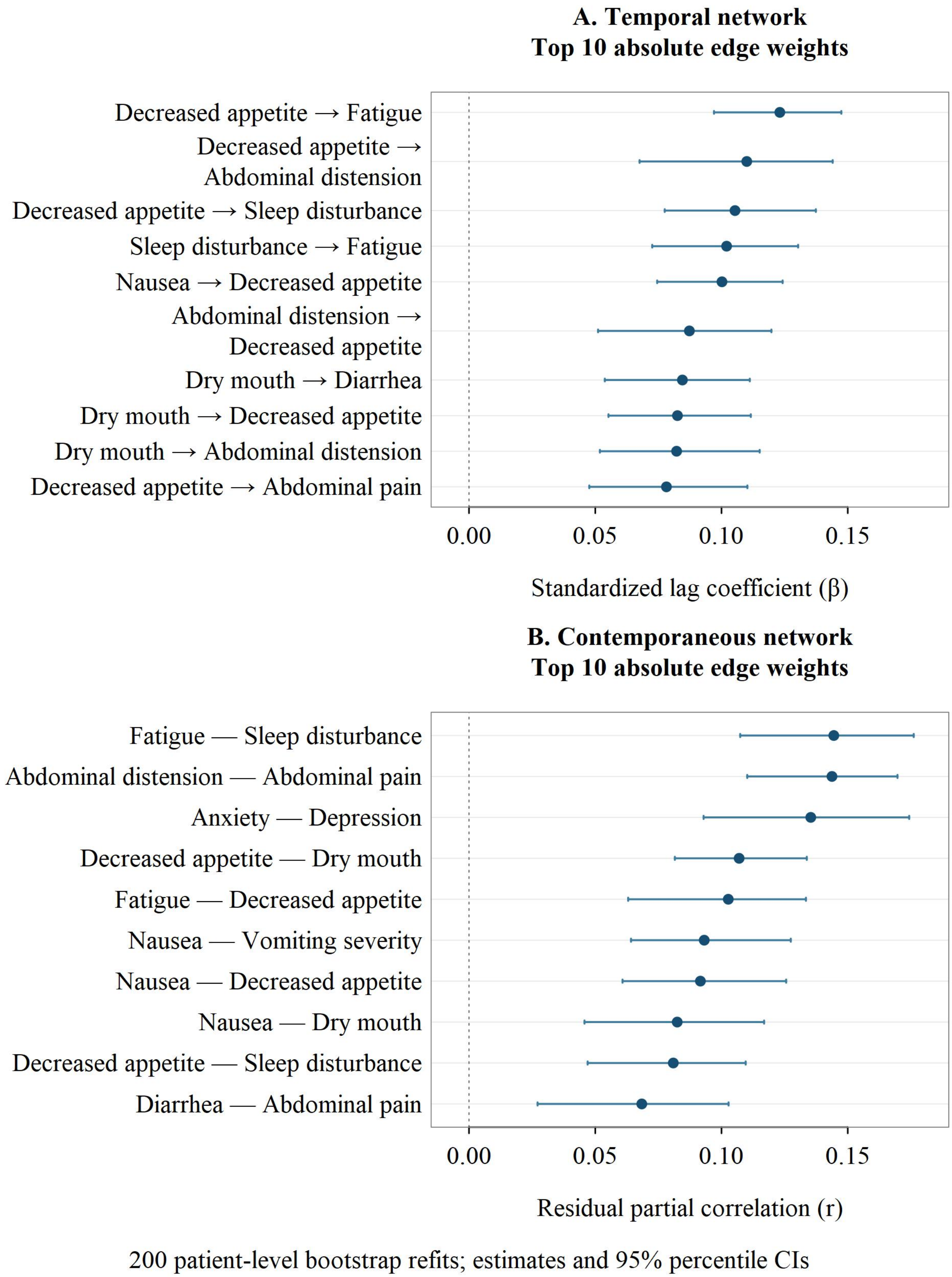

Figure S2 Patient-level bootstrap intervals for key network edges

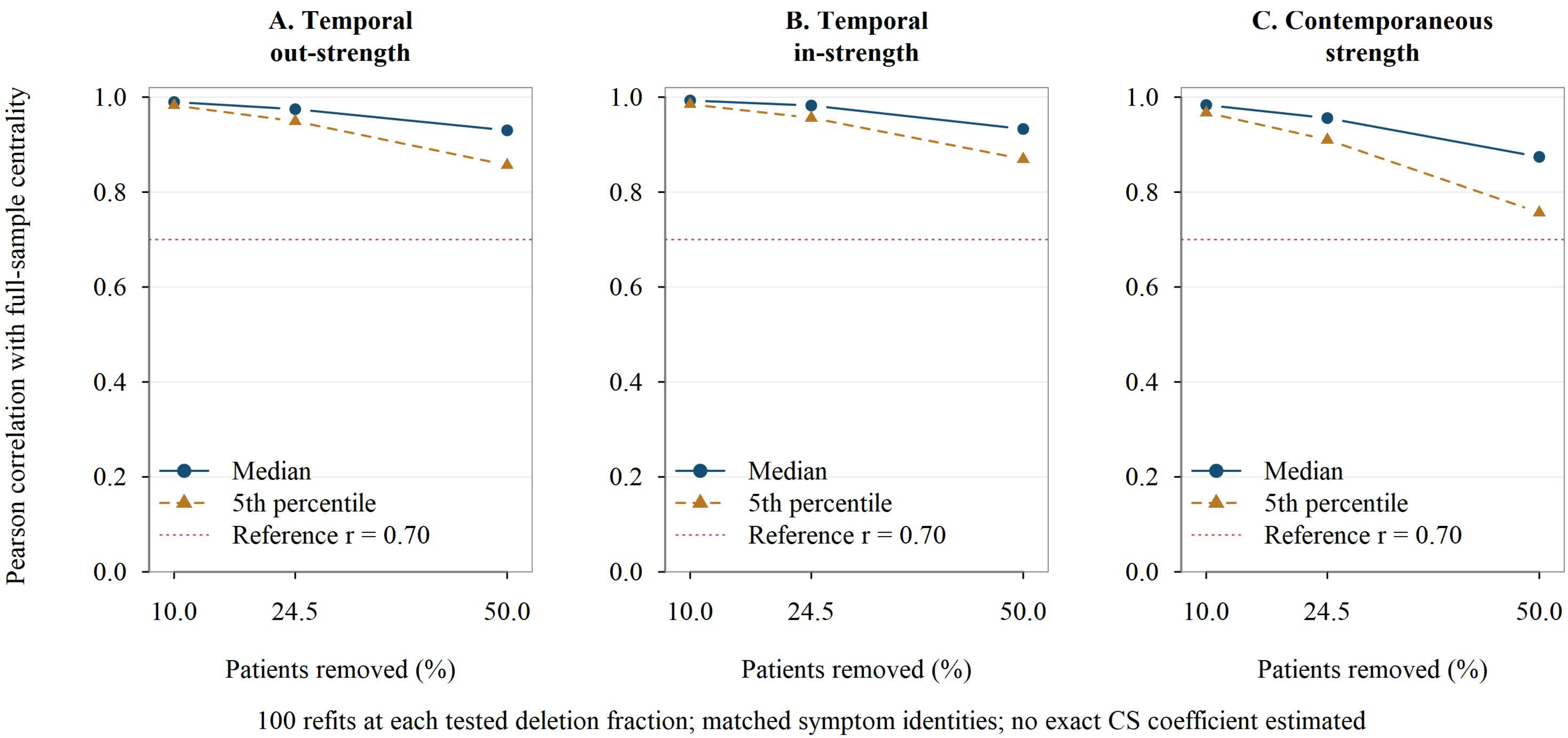

Figure S3 Centrality correlations after case dropping

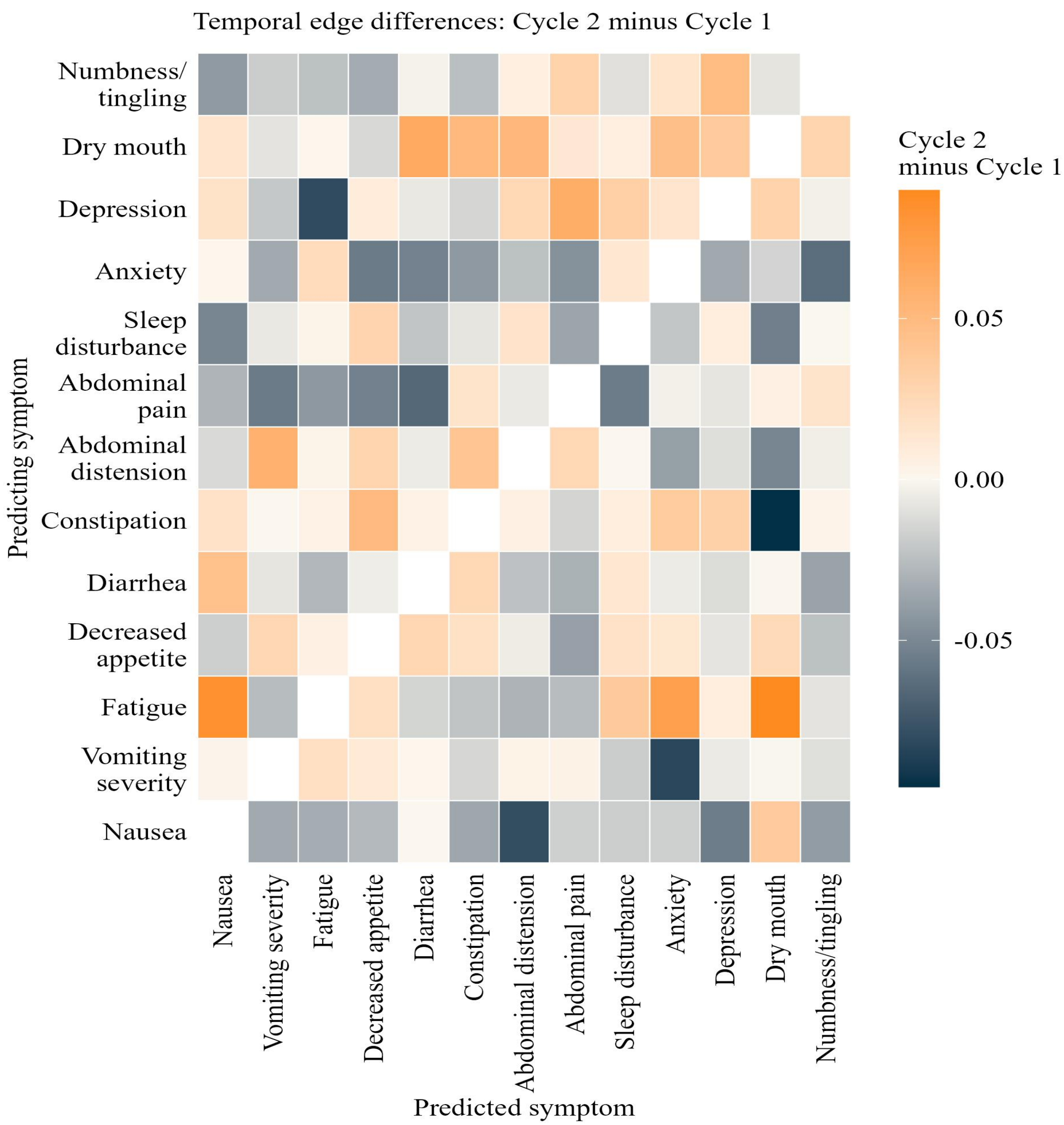

Figure S4 Heatmap of between-cycle differences in temporal edge weights

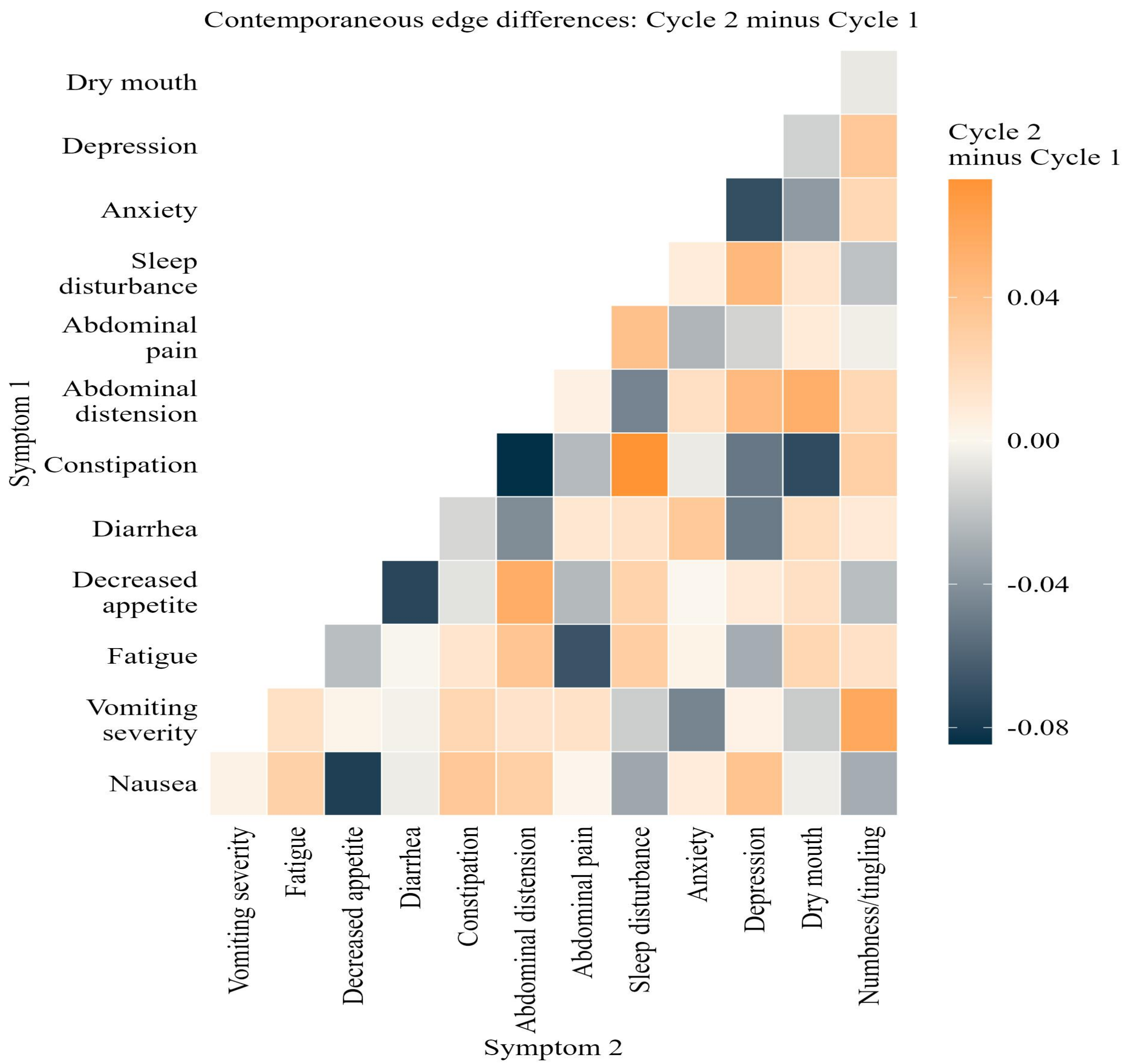

Figure S5 Heatmap of between-cycle differences in contemporaneous edge weights

Cells show contemporaneous partial correlations in cycle 2 minus those in cycle 1. The matrix is symmetric. Colors do not indicate statistical significance of the differences.

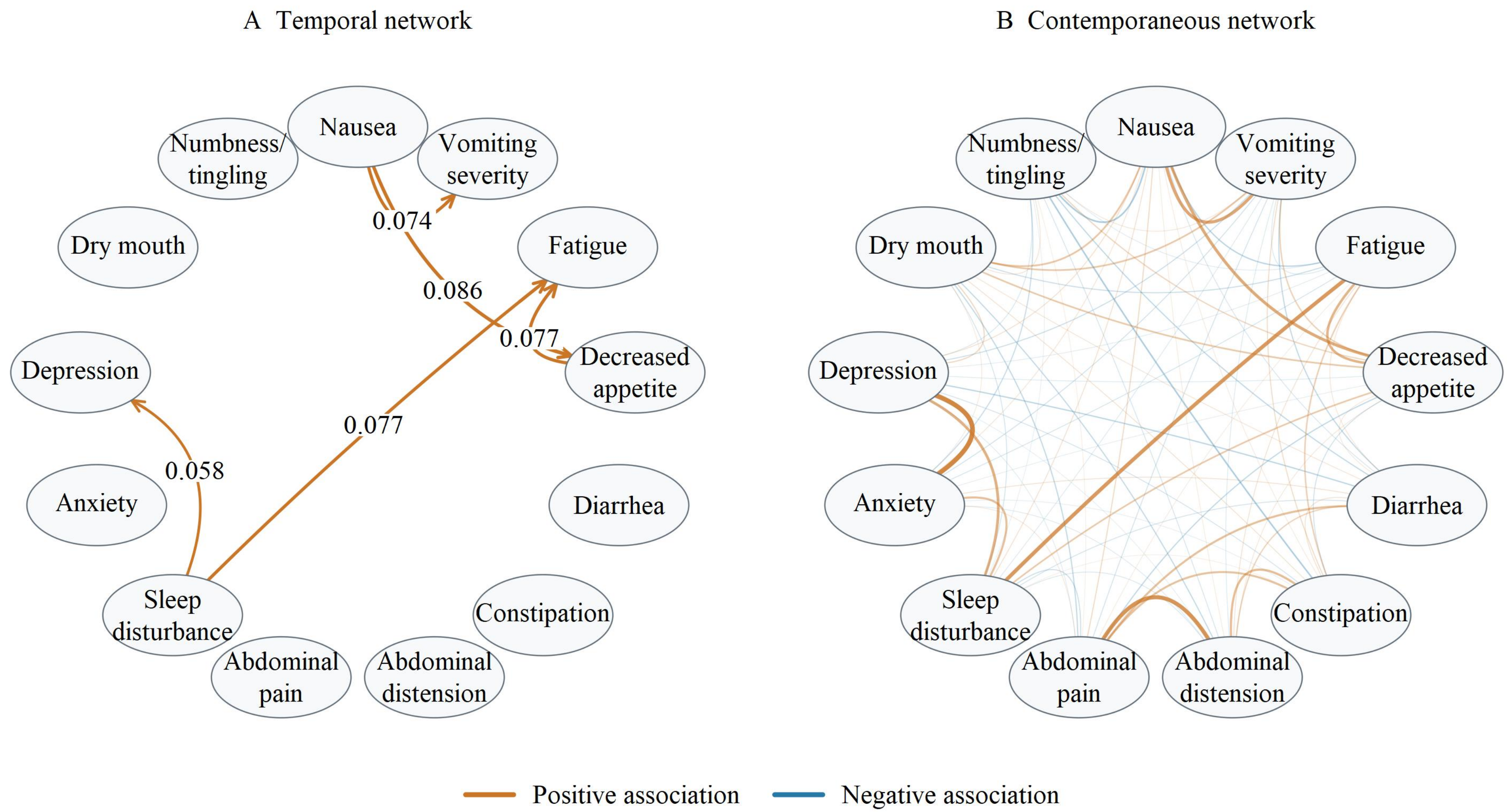

Figure S6 Networks after expanded clinical covariate residual adjustment. A: cross-symptom temporal edges with BH $q < 0.05$; autoregressive loops are omitted. B: all contemporaneous partial-correlation point estimates without significance filtering.

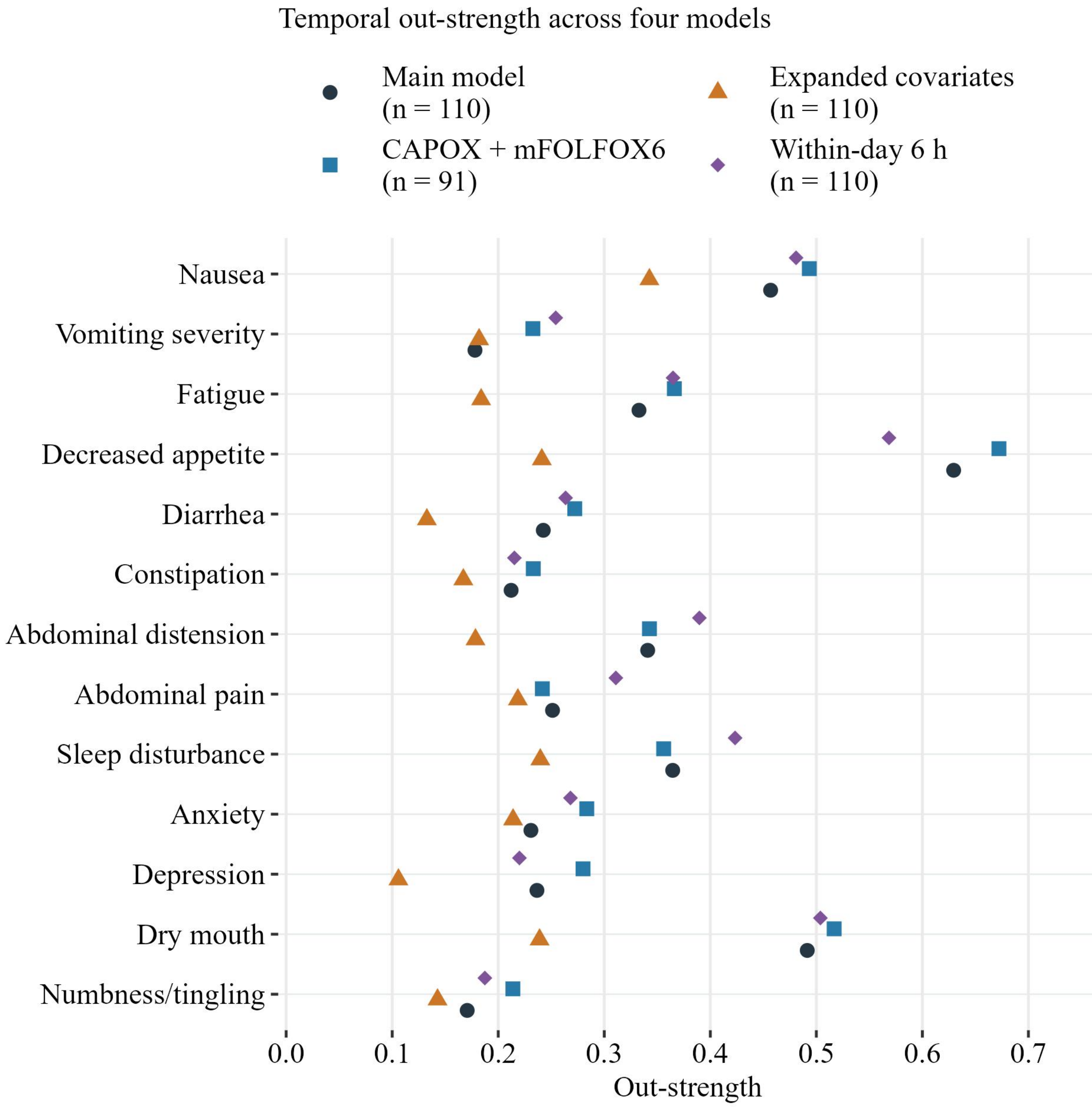

Sum of absolute coefficients to the other 12 symptoms; autoregression excluded; no P-value or BH filtering.

Fixed symptom identities and definitions; point estimates only, without confidence intervals.

Effective lag pairs: main, 3528; expanded covariates, 3354; CAPOX + mFOLFOX6, 2916; within-day 6 h, 2452.

Figure S7 Temporal out-strength across analyses

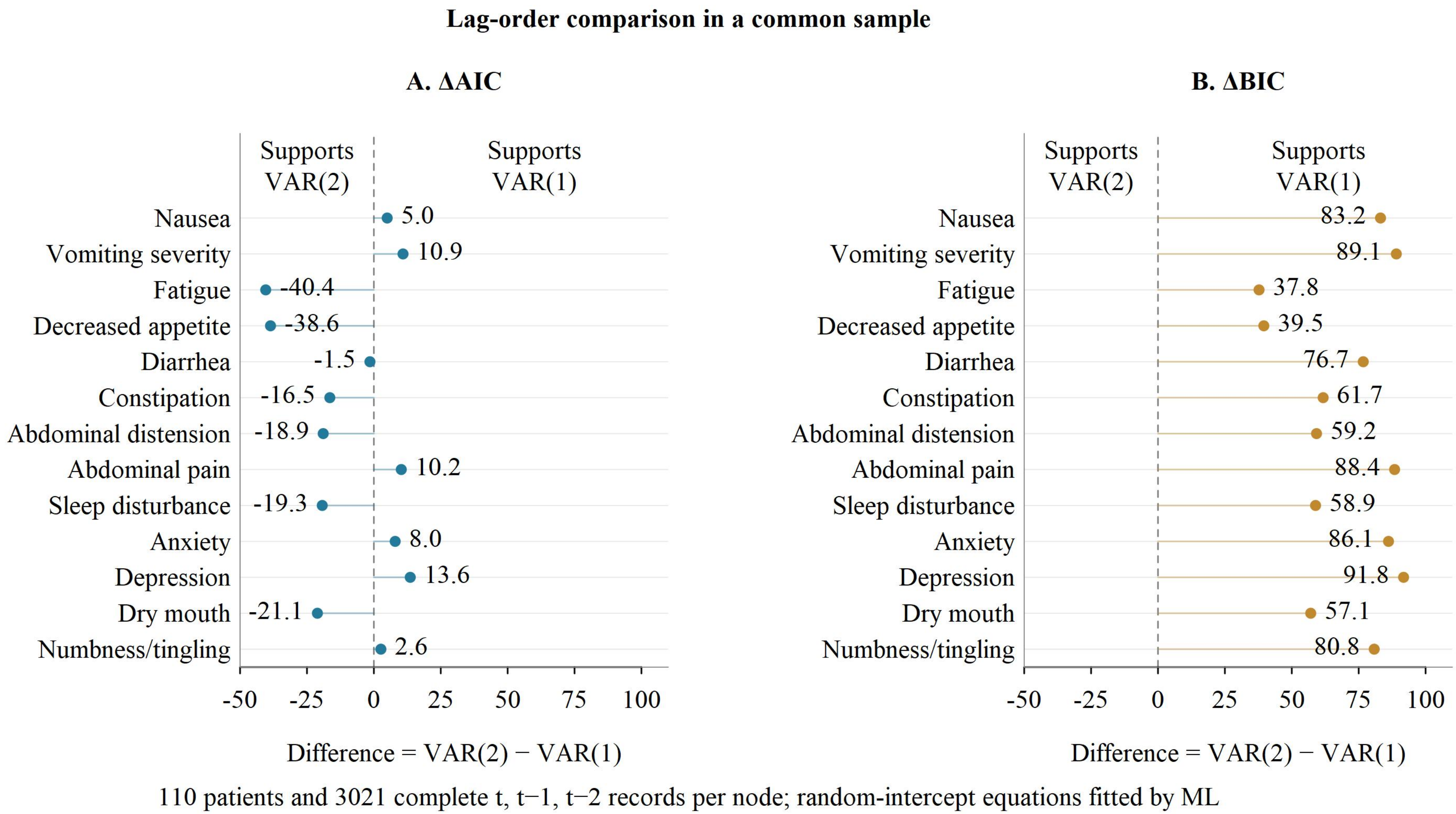

Figure S8 AIC and BIC comparisons of models with different lag orders

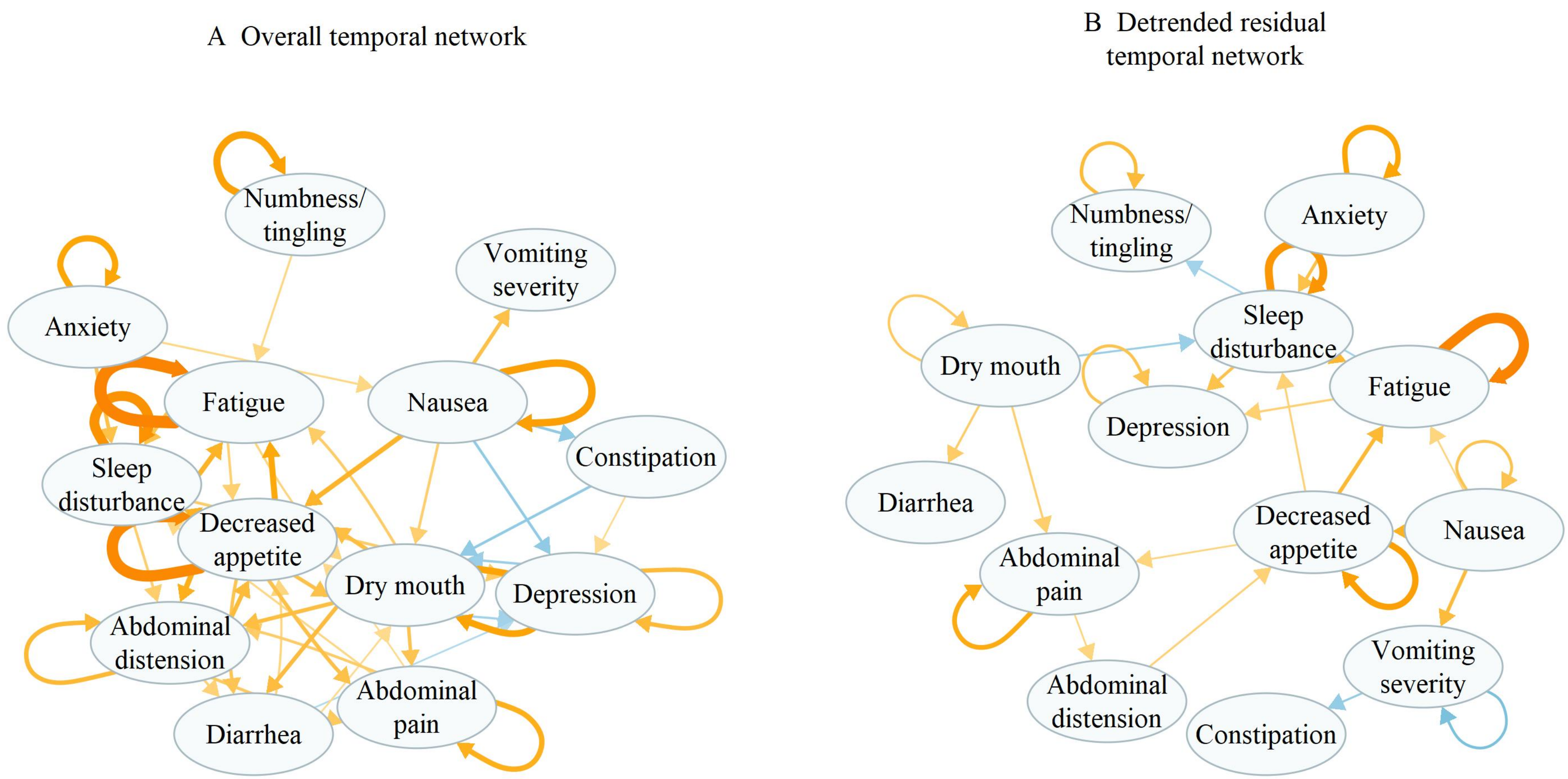

Figure S9 Overall and detrended temporal networks

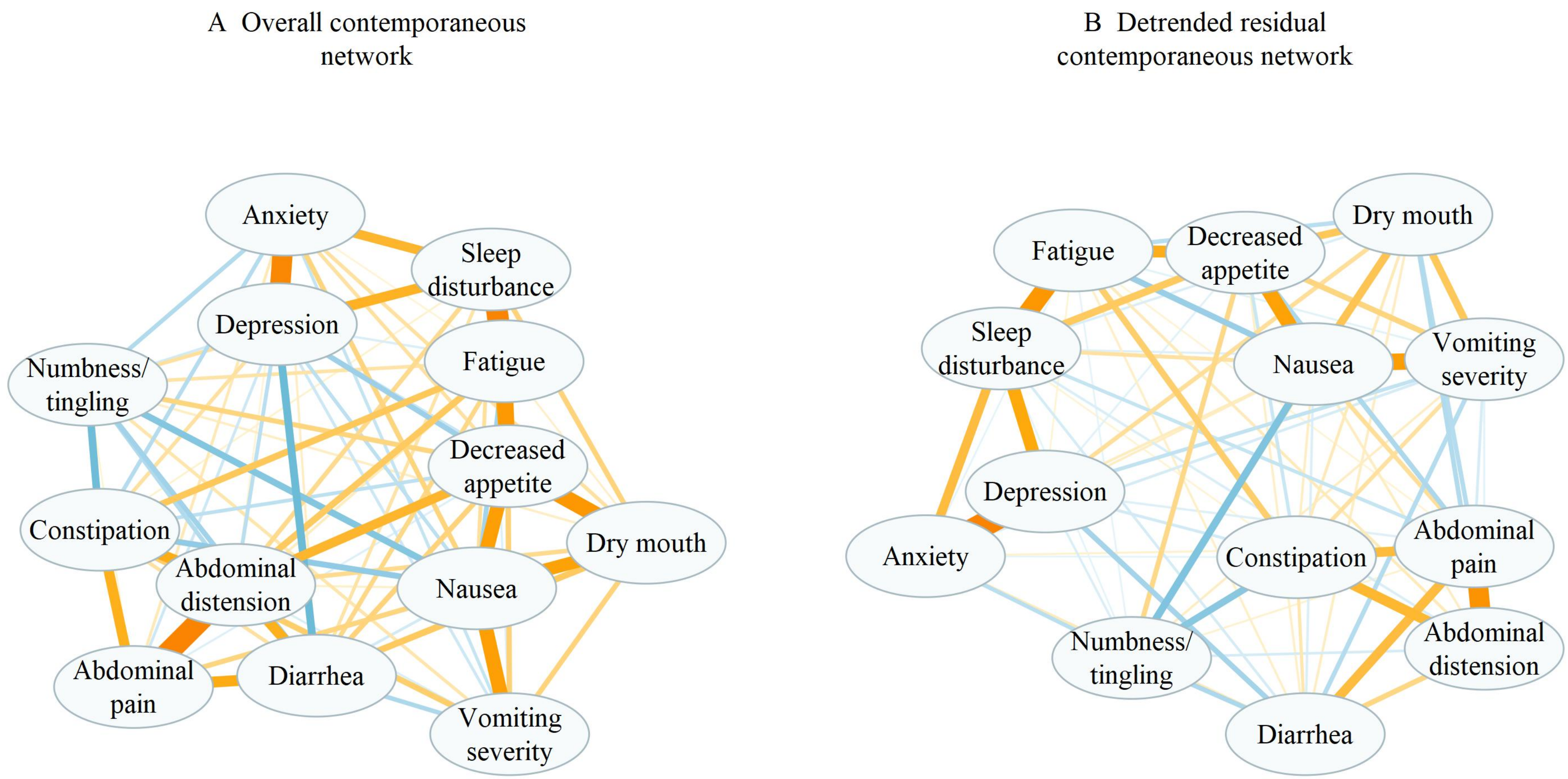

Figure S10 Overall and detrended contemporaneous networks
